# Guideline for the management of myasthenic syndromes

**DOI:** 10.1177/17562864231213240

**Published:** 2023-12-26

**Authors:** Heinz Wiendl, Angela Abicht, Andrew Chan, Adela Della Marina, Tim Hagenacker, Khosro Hekmat, Sarah Hoffmann, Hans-Stefan Hoffmann, Sebastian Jander, Christian Keller, Alexander Marx, Arthur Melms, Nico Melzer, Wolfgang Müller-Felber, Marc Pawlitzki, Jens-Carsten Rückert, Christiane Schneider-Gold, Benedikt Schoser, Bettina Schreiner, Michael Schroeter, Bettina Schubert, Jörn-Peter Sieb, Fritz Zimprich, Andreas Meisel

**Affiliations:** Department of Neurology with Institute of Translational Neurology, University Hospital Münster, Albert-Schweitzer-Campus 1, Building A1, Münster 48149, Germany; Friedrich-Baur-Institut an der Neurologischen Klinik und Poliklinik, LMU Munich, Munich, Germany; Universitätsklinik für Neurologie, Inselspital Bern, Bern, Switzerland; Klinik für Kinderheilkunde I, Universitätsklinikum Essen, Essen, Germany; Klinik für Neurologie, Universitätsklinikum Essen, Essen, Germany; Herzzentrum, Uniklinik Cologne, Cologne, Germany; Charité – Universitätsmedizin Berlin, Klinik für Neurologie mit Experimenteller Neurologie, Berlin, Germany; Klinik für Thoraxchirurgie, Krankenhaus Barmherzige Brüder, Regensburg, Germany; Klinik für Neurologie, Marien Hospital Düsseldorf, Düsseldorf, Germany; Department of Neurology with Institute of Translational Neurology, University Hospital Münster, Münster, Germany; Pathologisches Institut, Universitätsklinikum Mannheim, Mannheim, Germany; Facharztpraxis für Neurologie und Psychiatrie, Stuttgart, Germany; Klinik für Neurologie, Universitätsklinikum Düsseldorf, Düsseldorf, Germany; Kinderklinik und Kinderpoliklinik im Dr. von Haunerschen Kinderspital, LMU Munich, Munich, Germany; Klinik für Neurologie, Universitätsklinikum Düsseldorf, Düsseldorf, Germany; Chirurgische Klinik, Charité – Universitätsmedizin Berlin, Berlin, Germany; Neurologie, Katholisches Klinikum Bochum, Bochum, Germany; Friedrich-Baur-Institut an der Neurologischen Klinik und Poliklinik, LMU Munich, Munich, Germany; Klinik für Neurologie, Universitätsspital Zürich, Zürich, Switzerland; Klinik und Poliklinik für Neurologie, Uniklinik Cologne, Cologne, Germany; Deutsche Myasthenie Gesellschaft e.V., Bremen, Germany; Helios Hanseklinikum Stralsund, Stralsund, Germany; Universitätsklinik für Neurologie, AKH-Wien, Wien, Austria; Charité – Universitätsmedizin Berlin, Klinik für Neurologie mit Experimenteller Neurologie, Berlin, Germany

**Keywords:** congenital myasthenic syndromes, disease-modifying treatment, Lambert-Eaton myasthenic syndrome, myasthenia gravis, myasthenic syndromes, treatment guideline

## Abstract

Myasthenia gravis (MG), Lambert-Eaton myasthenic syndrome (LEMS), and congenital myasthenic syndromes (CMS) represent an etiologically heterogeneous group of (very) rare chronic diseases. MG and LEMS have an autoimmune-mediated etiology, while CMS are genetic disorders. A (strain dependent) muscle weakness due to neuromuscular transmission disorder is a common feature. Generalized MG requires increasingly differentiated therapeutic strategies that consider the enormous therapeutic developments of recent years. To include the newest therapy recommendations, a comprehensive update of the available German-language guideline ‘Diagnostics and therapy of myasthenic syndromes’ has been published by the German Neurological society with the aid of an interdisciplinary expert panel. This paper is an adapted translation of the updated and partly newly developed treatment guideline. It defines the rapid achievement of complete disease control in myasthenic patients as a central treatment goal. The use of standard therapies, as well as modern immunotherapeutics, is subject to a staged regimen that takes into account autoantibody status and disease activity. With the advent of modern, fast-acting immunomodulators, disease activity assessment has become pivotal and requires evaluation of the clinical course, including severity and required therapies. Applying MG-specific scores and classifications such as Myasthenia Gravis Activities of Daily Living, Quantitative Myasthenia Gravis, and Myasthenia Gravis Foundation of America allows differentiation between mild/moderate and (highly) active (including refractory) disease. Therapy decisions must consider age, thymic pathology, antibody status, and disease activity. Glucocorticosteroids and the classical immunosuppressants (primarily azathioprine) are the basic immunotherapeutics to treat mild/moderate to (highly) active generalized MG/young MG and ocular MG. Thymectomy is indicated as a treatment for thymoma-associated MG and generalized MG with acetylcholine receptor antibody (AChR-Ab)-positive status. In (highly) active generalized MG, complement inhibitors (currently eculizumab and ravulizumab) or neonatal Fc receptor modulators (currently efgartigimod) are recommended for AChR-Ab-positive status and rituximab for muscle-specific receptor tyrosine kinase (MuSK)-Ab-positive status. Specific treatment for myasthenic crises requires plasmapheresis, immunoadsorption, or IVIG. Specific aspects of ocular, juvenile, and congenital myasthenia are highlighted. The guideline will be further developed based on new study results for other immunomodulators and biomarkers that aid the accurate measurement of disease activity.

## Introduction

The German-language guideline for diagnosing and treating myasthenic syndromes has been updated by an expert panel in a process that started in 2019 and was completed in 2022.^1^ All disciplines involved in the care of myasthenic syndromes (e.g. neurologists, pediatricians, cardiologists, thoracic surgeons, geneticists, neuropathologists) were represented. The recommendations in this treatment guideline have been developed based on a consensus procedure with different recommendation levels (must/must not, should/should not, can be considered/waived) and consensus strengths (strong agreement, agreement, majority consent, no majority consent).

Essential changes to the previous guideline version are briefly summarized below.

## What’s new?

● The therapeutic goal is the best possible disease control while restoring the patient’s quality of life. Disease control can be divided into four levels:
  (1) full disease control without disease activity, no residual symptoms, freedom from disease activity;
  (2) full disease control with no detectable disease activity, but minimal residual symptoms with stability (incomplete remission);
  (3) incomplete disease control with disease activity: instability, deterioration, fluctuation with residual symptoms and continuous new or developing symptoms, ± fluctuations, ± crises;
  (4) no disease control with high disease activity [including ‘refractory’ myasthenia gravis (MG)]: continuous symptoms with or without crises or crisis-like deteriorations, resistance to therapy.

- For therapy and treatment decisions, the assessment of disease activity and progression is becoming increasingly important. In addition to the Myasthenia Gravis Foundation of America (MGFA) classification, this assessment is based on the (current) disease severity, which differentiates between a mild/moderate *versus* a (highly) active disease. The latter also includes the term ‘refractory generalized MG (gMG)’. Disease activity is determined by the severity of clinical symptoms, their duration, and tendency to regress, as well as clinical residuals and the presence or number of crisis-like exacerbations/crises.
- In addition to disease activity, therapy of MG is increasingly based on antibody (Ab) status with subtyping into acetylcholine receptor (AChR)-, muscle-specific receptor tyrosine kinase (MuSK), or lipoprotein-related protein 4 (LRP4)-positive and seronegative MG. While AChR-Ab belongs to the complement-binding immunoglobulin (Ig) G1 subclass and thus causes the complement-dependent destruction of the neuromuscular endplate, MuSK-Ab belongs to the non-complement-binding IgG4 subclass so that complement inhibitors are ineffective against this form.
- The phase III MGTX trial provided grade 1 evidence of long-term clinical improvement and steroid-sparing effect of thymectomy in patients aged 18–65 years with AChR-Ab-positive gMG of up to 5 years after first confirmatory diagnosis.^2,3^
- With the humanized monoclonal IgG-Ab eculizumab, the first C5 complement inhibitor was introduced as a therapy for MG in 2017. Results from the phase III REGAIN trial provided class 1 evidence leading to treatment approval in AChR-Ab-positive refractory gMG.^4^ Ravulizumab is another C5 complement inhibitor and was approved by the European Medicines Agency (EMA) in September 2022 for the indication of AChR-Ab-positive gMG in adults as an add-on to standard therapy based on positive results from the phase III CHAMPION trial.^5^
- Neonatal Fc receptors (FcRns) have been established as a further novel therapeutic target for the treatment of gMG. The Ab fragment efgartigimod has demonstrated safety and efficacy in the treatment of gMG in phase III ADAPT trial, with the primary end point of clinically meaningful improvement for Myasthenia Gravis Activities of Daily Living (MG-ADL) in AChR-Ab-positive MG.^6^ Following a positive vote of the EMA, the EU approved efgartigimod alfa in August 2022 as an add-on to standard therapy in the treatment of adult patients with gMG who are AChR-Ab-positive.^7^
- Other complement inhibitors, such as zilucoplan, and other modulators of FcRn, such as rozanolixizumab, have been tested in phase III trials, for which positive results are now available. These compounds differ primarily in the mode and frequency of administration.^6,8^ They are part of a therapeutic development that will significantly change the treatment of MG in the coming years.
- An important, although rare, differential diagnosis of autoimmune myasthenic syndromes – not only in children but also in adults – is the congenital myasthenic syndromes (CMS). The specifics of CMS and its diagnosis and therapeutic options are presented separately from the autoimmune myasthenic syndromes.

## Essential facts (1)

In the following, the most important recommendations of the current guideline are summarized.

● The diagnosis of MG is based on the history and physical findings of fatigable and fluctuating muscle weakness. The diagnosis is confirmed by positive findings in auto-Ab diagnostics and/or electrophysiology and/or pharmacological testing.
● The therapeutic goal is the best possible disease control while restoring or preserving the patient’s quality of life.
● In addition to the MGFA classification, assessment of disease course over time should be based on disease severity and disease activity categorized into mild/moderate *versus* (highly) active (including refractory).
● Disease activity should be defined based on the severity of clinical symptoms, duration, and regression tendency, taking into account clinical residuals and the presence or number of potentially life-threatening severe exacerbations or myasthenic crises. Fluctuations in clinical presentation or residual signs of disease should also be included.
For ongoing assessment of disease activity, severity, and response to MG-specific medications, clinical examinations including the MG-specific clinical scores must be performed at regular intervals and need to take into account the patient’s self-assessment. For this purpose, the Quantitative Myasthenia Gravis (QMG) Score, the MG-ADL, the MG-Quality of Life 15 (MG-QoL15r), and the current MGFA status should be applied.
● A (highly) active gMG (including ‘refractory MG’) can be defined as
(1) Moderate/high MGFA status (⩾MGFA IIb) and/or at least two recurrent severe exacerbations/myasthenic crises with the need for therapeutic intervention (IVIG, PE, and IA) within 1 year after diagnosis despite adequate disease-modifying and symptomatic therapy.
 or
(2) Persistent symptoms relevant to daily living (⩾MGFA IIa) and severe exacerbation/myasthenic crisis within the last calendar year despite adequate disease-modifying and symptomatic therapy.
or
(3) Persistent symptoms relevant to daily living, even of the mild/moderate course type (⩾MGFA IIa), for more than 2 years despite adequate disease-modifying and symptomatic therapy.
● Therapy must be based on age, thymic pathology, antibody status (AChR-Ab-, MuSK-Ab-, LRP4-Ab-positive as well as seronegative MG), and disease activity.
● For symptomatic therapy of MG, acetylcholinesterase inhibitors (AChE-I), primarily pyridostigmine, should be used.
● Oral glucocorticosteroids (GKS) should be used to treat mild/moderate to (highly) active gMG/juvenile MG (jMG) and ocular MG (oMG) as basic immunotherapeutics at doses appropriate to disease severity over the shortest possible period of time, taking into account comorbidities, contraindications, and side effects.
For AChR-Ab-positive, LRP4-Ab-positive, and seronegative gMG/jMG of mild/moderate activity, GKS and/or azathioprine (AZA; ± thymectomy) should be used as the first-choice therapy for disease course modification in addition to symptomatic therapy.
● As an alternative to AZA, mycophenolate mofetil (MMF), ciclosporin A (CSA), tacrolimus, or methotrexate (MTX) may be considered for second-choice treatment of gMG (to be used if AZA proves ineffective, is not tolerated, or is contraindicated). For second-choice treatment of jMG, MMF or tacrolimus may be considered as an alternative to AZA.
● For oMG, oral GKS ± AZA should be used as disease-modifying therapy in addition to symptomatic therapy. As an alternative to AZA, the use of MMF, CSA, tacrolimus, or MTX can be considered.
Symptomatic therapy of (highly) active MG, including ‘refractory to therapy’, should be supplemented by the following disease-modifying therapies: In AChR-Ab-positive status, complement inhibitors (eculizumab, ravulizumab) or FcRn modulators (efgartigimod) ± thymectomy should be selected. In AChR-Ab-positive, LRP4-Ab-positive, or seronegative status, CD20 antibody depletion (rituximab) ± thymectomy may be considered as first-choice therapy for disease course modification. Second-choice treatments are IVIG and plasmapheresis (common abbreviations: PLEX and PE; PE will be used hereafter)/immunoadsorption (IA). For generalized jMG, IVIG/PE should be selected as first-choice therapies, and rituximab and eculizumab as second-choice therapies. Efgartigimod and ravulizumab may be considered as second-choice therapies for AChR-Ab-positive jMG.
● In the event of an impending and manifest myasthenic crisis, rapid admission and treatment should be provided in a surveillance or intensive care unit with experience in neuromuscular diseases. IVIG or PE/IA should be selected as treatment in this situation.
● For MuSK-Ab-positive MG with mild/moderate activity, symptomatic therapy with AChE-I must be complemented by GKS ± AZA as first-choice therapy for disease course modification. Rituximab must be selected for patients with (highly) active disease course (including ‘refractory to therapy’). Second-choice therapies must be selected analogous to AChR-Ab-positive MG while FcRn modulators (efgartigimod) may also be considered.
● Juvenile gMG and oMG should be treated with drugs in the same way as adult MG – according to Ab subtype and disease activity. In prepubertal children, spontaneous remissions should be considered. Therapy with rituximab and eculizumab in juvenile gMG should also be discussed individually as an off-label option. In a crisis, PE/IA can be considered, and the administration of IVIG can be considered.
● Every MG patient should be evaluated for the presence of a thymoma using thoracic computed tomography (CT) or magnetic resonance imaging. If a thymoma is suspected, the thymus including thymoma should be surgically removed as completely as possible. Depending on the histopathological findings, further therapies such as radio- and/or chemotherapy may be necessary. Even in children and adolescents, a thymoma – despite its rarity – should be excluded based on image morphology.
● For non-thymoma-associated MG, the following applies with regard to thymectomy:
(1) In patients with AChR-Ab-positive gMG aged 18–65 years, thymectomy (transsternal or minimally invasive) should be performed as early as possible within 2 years and no later than 5 years after confirmed diagnosis.
(2) Thymectomy may also be considered in seronegative gMG and LRP4-Ab-positive gMG with high disease activity during the first 2 years of disease, if possible.
(3) MuSK-Ab-positive MG patients should not undergo thymectomy.
(4) Thymectomy may also be considered in generalized AChR-Ab-positive jMG and decided on an individual basis.
(5) In children and adolescents aged 5–12 years, thymectomy should be performed only after failure of drug therapy (AChE-I, GKS). In adolescents aged 13 years and older, the procedure should be continued as in (1).
● Vaccinations, including COVID-19 vaccinations, should be administered as usual in MG including jMG according to recommendations of national competent authorities. This especially applies to live vaccinations and the indication for vaccination in jMG. Prior to therapy with complement inhibitors, vaccination against meningococcal serogroups A, C, Y, W135 (1 × Menveo^®^), and B (2 × Bexsero^®^ 4 weeks apart) must be performed. Live vaccinations should not be given during immunotherapy. If time permits, required vaccinations should be completed at least 4 weeks prior to initiation of immunotherapy. In the case of rituximab therapy, vaccinations should be given 1 month before a planned administration or at least 1 month after rituximab therapy or before the next administration.
● In case of pregnancy and a desire to have children, a specific consultation should be made in a center specialized in myasthenia.
In Lambert-Eaton myasthenic syndrome (LEMS), symptomatic therapy should be with amifampridine (3,4-diaminopyridine, 3,4-DAP) and pyridostigmine. In paraneoplastic LEMS (pLEMS), tumor treatment should be performed as usual. GKS should be used for the treatment of LEMS as a basic immunotherapeutic agent at a dosage appropriate to the severity of the disease for as short a period as possible, taking into account comorbidities, contraindications, and side effects. AZA should be used for steroid-sparing therapy. As an alternative to AZA, the use of MMF, CSA, tacrolimus, or rituximab may be considered. MTX should be avoided, especially in pLEMS. IVIG or, if necessary, plasmapheresis should be used to treat a crisis-like deterioration.
● In Ab-negative myasthenia, the differential diagnosis of CMS should be considered depending on the response to immunotherapy. If CMS is suspected, molecular genetic diagnostics should be performed to confirm the diagnosis and plan therapy.
● Multimodal (pharmacologic and nonpharmacologic) therapy (including thymectomy) of myasthenic syndromes should be performed in or in close coordination with myasthenia centers.

## Essential recommendations (2)

In this section, all recommendations are presented with brief explanations or rationales.

### Assessment of disease severity, activity, and progression (2.1)

**Table:** 

**Recommendation 2.1a**
The therapeutic goal for MG *must* be the best possible disease control with the best possible preservation or restoration of quality of life. Achievement of disease control *should* be graded according to the following four levels: (1) Full disease control with no disease activity and no residual symptoms. (2) Full disease control with no detectable disease activity, but minimal residual symptoms with stability (incomplete remission). (3) Incomplete disease control with disease activity: instability, deterioration, fluctuation with residual symptoms and continuous new or developing symptoms, ± fluctuations, ± crises. (4) No disease control with high disease activity (including refractory MG): continuous symptoms with or without crises or crisis-like deteriorations, resistance to therapy. Assessment of disease progression *should* be based on disease severity and disease activity categorized into mild/moderate *versus* (highly) active (including refractory MG), in addition to the classification of the MGFA. The definition of disease activity *should* be based on the severity of clinical symptoms/duration/tendency to regress in conjunction with clinical residuals and the presence or number of crisis-like exacerbations/crises. Fluctuations in clinical presentation in terms of a marker for detectable or residual signs of disease *should* also be included.
Strong agreement

The definition of MG activity is based on:

- the severity of myasthenic symptoms
- the time course of MG, and
- the response to symptomatic and disease-modifying therapy including thymectomy (except MuSK-positive MG).

Valid biomarkers are not yet available. For example, a recent systematic literature review found a variable or limited correlation between autoantibody levels and disease activity in patients with MG. However, the quality of available studies is insufficient to draw definitive conclusions. Therefore, routine clinical use of autoantibody testing to determine disease activity cannot be recommended.^9^ Hence, decisions for therapeutic practice, especially the choice of disease-modifying therapy, are based on disease activity. In the following, a distinction is made between mild/moderate and (highly) active and refractory MG. A review of relevant literature combined with an expert discussion resulted in the following definition for ‘(highly) active MG’ (which also includes refractory MG):

(1) Persistent symptoms relevant to daily living (⩾MGFA IIb) and/or at least two recurrent severe exacerbations/myasthenic crises with the need for therapeutic intervention (IVIg, PE, and IA) within 1 year of diagnosis despite adequate disease-modifying and symptomatic therapy.
or
(2) Persistent symptoms relevant to daily living (⩾MGFA IIa) and severe exacerbation/myasthenic crisis within the past year despite adequate disease-modifying and symptomatic therapy.
or
(3) Persistent mild/moderate symptoms relevant to daily living (⩾MGFA IIa) for more than 2 years despite adequate disease-modifying and symptomatic therapy.

*Note*: The assessment of severity is based on the MGFA classification. However, the MGFA status used here only takes into account the severity at the time of clinical assessment and not the highest severity ever reached during the course of the disease.

In about 10% of MG patients, a satisfactory therapeutic response is not achieved.^10,11^ For this group, the term ‘refractory myasthenia’ has been coined, which in clinical practice is difficult and used rather inconsistently, but has been officially implemented in particular in the context of the approval text for eculizumab therapy (‘refractory AChR-Ab-positive gMG’).^12^ Definitions of refractory myasthenia include the following aspects: severe myasthenia, failure to respond to immunotherapy of sufficient dose and duration, discontinuation of therapy due to drug side effects, implementation of escalated therapy measures such as IVIg administration, and/or the need for intensive care (myasthenic crisis).^13,14^

*Disease severity, progression, activity, and response to disease-modifying therapies* are determined by examinations depending on the symptoms expression at least once every 6 months using scales and scores (Supplemental Table 1). *Principally, the classifications are not necessarily categorical or static and require review and continuous monitoring*.

**Table:** 

**Recommendation 2.1b**
For ongoing assessment of disease activity and severity and response to MG-specific medications, clinical medical examinations including the collection of clinical scores *must* be performed at regular intervals and need to take into account the patient’s self-assessment (e.g. QMG Score, MG-ADL, MG-QoLr).
Strong agreement

### Therapy (2.2)

**Table:** 

**Recommendation 2.2a**
Therapy *must* be based on age, thymic pathology, Ab status (AChR-Ab-, MuSK-Ab-, LRP4-Ab-positive as well as seronegative MG), and disease activity.
Strong agreement

**Figure 1.**
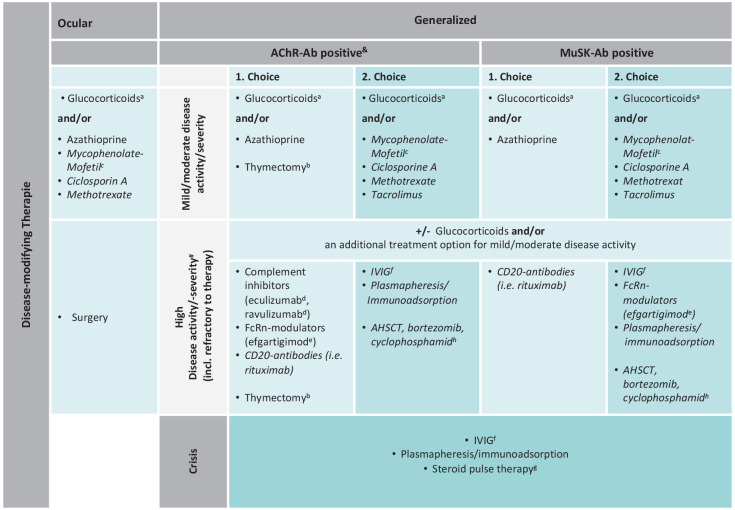
Scheme for the disease-modifying therapy of MG (therapy scheme). #: A (highly) active generalized MG (including ‘therapy refractory’ MG) may be defined as (a). moderate/high MGFA score (⩾MGFA IIb) and/or at least two recessive severe exacerbations/myasthenic crises requiring therapeutic intervention (IVIG, PLEX, and IA) within 1 year after first diagnosis and despite adequate disease-modifying and symptomatic treatment or (b). persistent symptoms relevant to daily living (⩾MGFA IIa) and one severe exacerbation/myasthenic crisis within the last 12 months despite adequate disease-modifying and symptomatic treatment or (c). persistent symptoms relevant to daily living (⩾MGFA IIa) present in MG of mild/moderate disease course despite adequate disease-modifying and symptomatic treatment for more than 2 years. *Note*: Disease severity is assessed according to MGFA classification. However, the MGFA status used here only takes into account the severity at the time of clinical assessment and not the highest score ever assessed in the course of disease. &: Seronegative and LRP4 antibody-positive MG are generally treated like AChR-Ab-positive MG. In italics: *Off-label* therapies (a). Steroids are not indicated as long-term therapy (at least above Cushing’s threshold, e.g. for prednisolone 7.5 mg/day); steroid-sparing strategies should be applied at an early stage. (b). Consider age (usually 18–65 years) and disease duration (usually <5 years); obligatory in case of suspected thymoma. (c). Off-label use of MMF is reimbursable as second choice therapy in Germany. (d). Eculizumab is on-label for the treatment of refractory AChR-Ab-positive gMG, ravulizumab is approved as add-on therapy for AChR-Ab-positive gMG. (e). Efgartigimod is approved as an add-on therapy for AChR-Ab-positive gMG. (f). IVIG is refundable when used as an off-label treatment for severe myasthenic exacerbations; SCIG can be used instead of IVIG in exceptional cases, but reimbursement is not regulated for this indication in Germany. *(g). Cave*: Steroid dip. (h). Justifiable as expanded access/compassionated use. Source: Own illustration. Ab, antibody; AChR, acetylcholine receptor; gMG, generalized MG; MG, myasthenia gravis; MGFA, Myasthenia Gravis Foundation of America; LRP4, lipoprotein-related protein 4; MMF, mycophenolate mofetil; SCIG, subcutaneous Ig.

In addition to symptomatic therapy, patients diagnosed with MG must be offered a disease-modifying (immuno)therapy (Figure 1), provided that therapy support is given by (i) adequate infrastructure, (ii) adequate disease assessment, (iii) continuous monitoring of the disease but also the therapy, and (iv) knowledge, recognition as well as treatment of therapeutic side effects.

The selection of the optimal disease-modifying therapy, based on knowledge of the respective mechanism of action, follows two main treatment approaches. They are based on the evaluation of the risk of further MG progression and risks *versus* efficacy of disease-modifying therapies. The distinction is made between

(1) Starting treatment with lower-potency medications with long-established and relatively safe risk profiles. Escalating to more potent medications if further disease activity is detected. This is currently the most common approach in myasthenia therapy and is recommended for mild/moderate MG.
(2) Starting treatment with a medication of higher efficacy, recommended for (highly) active disease courses, early after diagnosis if necessary.

In first-choice treatment, the auto-Ab status has no influence so far on the drug therapy decisions. Thus, in mild/moderate MG, a stepwise regimen with AChE-I, GKS, and AZA is initially recommended. In the case of incomplete disease control after first-choice treatment or already initially (highly) active MG, Ab-specific treatment concepts are used increasingly and also applied earlier – in individual cases also as first-choice therapy.

In children and adolescents with jMG, the specificities of age and courses must be considered in therapeutic decisions. This concerns higher remission rates (15–39%) and frequently isolated ocular forms in prepubertal children (26–38%).^15–17^

#### Problems with *off-label* use

Only a selection of pharmaceuticals that have been successfully used in practice for many years are officially approved for MG therapy. These restrictions on the free choice of therapy must not result in patients being deprived of a potentially effective therapy. Based on current case law, patients must be made aware of off-label use of a drug prior to treatment and the need for payer approval. It is therefore advisable to record all information on indications and typical side effects of unapproved drugs in writing and attaining the patient’s signature. Off-label drug use can be justified by the fact that MG is a rare, serious chronic disease that permanently impairs quality of life with potentially life-threatening exacerbations, that the efficacy of the off-label therapy options has been tested in scientific studies, that there is no alternative therapy, and that there is a reasonable prospect of successful treatment with the preparations used based on the data available [decision of the Federal Social Court (Case No. B1KR7/05R)]. Off-label use in MG is plausible under two main conditions:

(1) Contraindications to or intolerance of approved substances (prednisolone, AZA).
(2) Inadequate response to AZA and/or long-term steroid requirements greater than 7.5 mg prednisolone equivalent/day (d) (‘Cushing’s threshold’).

### Symptomatic therapy (2.3)

**Table:** 

**Recommendation 2.3a**
For symptomatic therapy of MG, AChE-I, predominantly pyridostigmine, *should* be used. Pyridostigmine *must* be used as a symptomatic therapy for all forms of MG in immediate-release and/or sustained-release form depending on disease severity, concomitant diseases, side effects, and individual therapeutic doses ranging up to 720 mg. Doses above 720 mg p.o. are tolerated only in exceptional cases. In children and adolescents, attention *must* be paid to a weight-adapted dose.
Agreement

**Table:** 

**Recommendation 2.3b**
Administration of ambenonium, neostigmine, or distigmine *may be considered* in patients with intolerance to or ineffectiveness of pyridostigmine.
Strong agreement

AChE-I represents the most important symptomatic therapy. The efficacy of these substances has been proven by electrophysiological studies. However, only a small proportion of patients (<20%) with gMG are clinically stable in the long term with purely symptomatic therapy.^18^ 3,4-DAP is primarily used in LEMS. Except for a negative placebo-controlled trial for the use of 3,4-DAP in children and adolescents, no systematic data exist on its efficacy in autoimmune MG. It is therefore reserved for patients who do not respond adequately to other symptomatic therapies but whose condition improves under this regimen. This applies, among others, to MuSK-Ab-positive MG (Supplemental Table 2).

### Immunotherapy (2.4)

GKS and/or immunotherapy should be used in all patients with myasthenia who are insufficiently treated despite an adequate trial of pyridostigmine.^19^ Several retrospective cohort studies suggest that patients with initially pure ocular myasthenia are less likely to develop progression to gMG under immunotherapy.^20–22^ Study-based experience and prognostic parameters for termination of immunotherapy are sparse.^23^ After several years of stable remission, a protracted discontinuation attempt can be made.

Abrupt discontinuation of immunotherapy in an insufficiently stabilized state is risky and should be avoided as it may lead to the recurrence of myasthenic symptoms and can escalate to a myasthenic crisis.^23,24^ Performing a thymectomy reduces the required dose of immunotherapeutics over time and thus increases the chance that immunotherapeutics can be dispensed with in the long term.^2,3^

Opportunistic infections, including progressive multifocal leukoencephalopathy, are more likely with increased duration of immunotherapy. The risks of the different immunotherapeutics vary and must be considered accordingly. Malignancies may also occur, so therapy with AZA over more than 10 years should generally be avoided and the maximum dose for cumulative agents (e.g. cyclophosphamide) should not be exceeded. Monitoring and adjustment of this therapy should be done in consultation with a specialty outpatient clinic. The goal is full or extensive remission, which often can only be obtained with continuous immunotherapy. Therefore, depending on MG activity, appropriate changes in therapy are necessary. The contraindications of childbearing and pregnancy must be taken into account. If individual decisions deviating from this approach are made for compelling clinical reasons, it is recommended to document these in writing. In addition, a standardized explanation of the off-label use – also taking into account age – as well as the risks and benefits of the respective immunotherapy should be provided and a written declaration of consent should be obtained from the patient before treatment start. It is important to assess the vaccination history before starting a prolonged immunotherapeutic medication and to complete outstanding vaccinations if possible.

### Therapy for mild/moderate MG (2.4.1)

**Table:** 

**Recommendation 2.4.1a**
Oral GKS as basic immunotherapeutics *must* be used to treat mild/moderate to (highly) active gMG/jMG and oMG at doses appropriate to disease severity over the shortest possible period of time, taking into account comorbidities, contraindications, and side effects.
Strong agreement

**Table:** 

**Recommendation 2.4.1b**
For AChR-Ab-positive, LRP4-Ab-positive, and seronegative gMG/jMG* of mild/moderate activity, GKS and/or AZA (± thymectomy) *must* be used as a first-choice disease-modifying therapy in addition to symptomatic therapy. As an alternative to AZA, treatment with MMF, CSA, tacrolimus, or MTX *may be considered* as second-choice for gMG (to be used in case of inefficacy, lack of tolerability, and contraindications). As an alternative to AZA, treatment with MMF or tacrolimus may be considered as second-choice for jMG. *For MuSK-Ab-positive MG, see recommendation 2.4.4a
Strong agreement

**Table:** 

**Recommendation 2.4.1c**
For oMG, GKS ± AZA *must* be used as disease-modifying therapy in addition to symptomatic therapy. Alternative to AZA, the use of MMF, MTX, tacrolimus as well as CSA *can be considered*.
Strong agreement

GKS as monotherapy or in combination with AZA are first-choice agents for immunotherapy.^25^ Other immunotherapies should be considered as second-choice treatment for MG with increased disease activity and can be used when contraindications, intolerance, or inadequate response to adequate doses of standard therapy apply.

Second-choice agents include, in alphabetical order, CSA,^26^ MTX,^27^ MMF,^19,28,29^ and tacrolimus.^30^ Data comparing the efficacy of the various immunotherapeutics are scarce. The often long latencies between therapy initiation and clinical onset of action must be taken into account.

#### Azathioprine

AZA is the most frequently used immunotherapeutic agent in MG treatment besides GKS^31–34^ and has been approved for this purpose in Germany since 2004 based on a positive randomized-controlled trial.^25^ Due to the slow onset of action, therapeutic success with monotherapy is not to be expected before several months have elapsed. The therapy should be started with 25–50 mg daily in the first week and then gradually increased over 3–4 weeks under regular laboratory control. Alternatively, after a trial dose (of e.g. 50 mg daily for 3 days), a target dose of 2–3 mg/kg bw daily can be started more rapidly. An absolute lymphocyte count of 600–1000/μl is targeted during therapy.

#### Ciclosporin A

CSA was effective in a placebo-controlled study of class 1 evidence.^26^ In contrast to the study treatment setup (CSA monotherapy, high dosage 6 mg/kg bw), CSA is now either used in combination with GKS or, in case of GKS contraindications (such as diabetes mellitus), at a lower dosage (initially at 3–4 mg/kg bw, subsequently at 2–2.5 mg/kg bw, divided into two daily doses).

#### Methotrexate

A comparative study with MTX (17.5 mg/week) in 24 patients with gMG produced the same steroid-sparing effect as AZA (2.5 mg/kg bw/day) over an observation period of 2 years.^27^ By contrast, a placebo-controlled study over 12 months failed to demonstrate a steroid-sparing effect of 20 mg MTX (p.o.).^35^ MTX can be administered as a drug of the reserve, in concordance with its use in rheumatoid arthritis, in a dosage range of 7.5–25 mg s.c./i.v./i.m. once a week with a folic acid (5 mg) rescue 24–48 h after MTX administration.

#### Mycophenolate mofetil

In two phase III studies, neither an advantage of MMF over monotherapy with prednisone as initial therapy^29^ nor a steroid-sparing effect over a period of 12 and 36 weeks, respectively,^29,36^ could be seen. Long-term open-label follow-up data gave evidence of a positive effect of MMF on clinical course as well as a steroid-sparing effect.^28^ In Germany, MMF is prescribed off-label for MG as a second-choice agent.

#### Tacrolimus

The side effect profile is comparable to that of CSA and strongly dose dependent. Tacrolimus was developed in Japan and is approved there for the treatment of MG.^37,38^ Two controlled trials could not confirm a steroid-sparing effect of tacrolimus after 6 and 12 months, but a post hoc analysis of the results suggested a benefit in terms of improvement of quantitative myasthenia scores.^30,39^

All treatment options for mild/moderate MG and all intensified therapy options are summarized in Supplemental Table 3.

### Intensified therapy (2.4.2)

**Table:** 

**Recommendation 2.4.2a**	**New** [2022]
Symptomatic therapy of (highly) active including refractory MG should be supplemented by the following disease-modifying therapies: • In AChR-Ab-positive status, complement inhibitors (eculizumab^i^, ravulizumab^i,vi^) or FcRn modulators (efgartigimod^ii,vi^) ± thymectomy *must* be used. • In MG with an LRP4-Ab-positive^iii^ or seronegative^iii^ status, complement inhibitors or FcRn modulators (efgartigimod^ii^) ± thymectomy *may be considered*. • In AChR-Ab-positive, LRP4-Ab-positive, or seronegative status, CD20 antibody depletion (rituximab)^iv^ ± thymectomy *may be considered* as first-choice therapy for disease modification. The use of complement inhibitors is warranted only in the presence of evidence of progression with a complement-dependent mechanism. Second-choice drugs *should* be IVIg and PE/IA. In individual cases, other agents or procedures such as autologous haematopoietic stem cell therapy (AHSCT), bortezomib, and cyclophosphamide *may also be considered*. In jMG^v^, IVIg/PE *must* be used as first-choice therapies and rituximab and eculizumab as second-choice therapies. Efgartigimod^vi^ and ravulizumab^vi^ *may be considered* second-choice therapies. ^i^Eculizumab is approved only for refractory AChR-Ab-positive gMG, whereas ravulizumab is approved as an add-on therapy for AChR-Ab-positive gMG. ^ii^Efgartigimod is approved as an add-on therapy only for AChR-Ab-positive gMG. ^iii^Eculizumab, efgartigimod, and ravulizumab are *off-label* in this indication. ^iv^Rituximab is *off-label* in this indication. ^v^Eculizumab, efgartigimod, ravulizumab, and rituximab are *off-label* in this indication. ^vi^Not permitted in Switzerland.	
Strong agreement	

#### Rituximab

Rituximab is a monoclonal chimeric Ab that specifically binds to the marker CD20 on the membrane of pre-B and mature B lymphocytes and causes transient B-cell depletion (usually for 3–9 months). In rare cases, prolonged depletion of circulating B cells may occur for a year or longer. There is currently no approval for the treatment of MG. However, a large number of independent case series exist that report successful use of rituximab, in patients with MG, particularly MuSK-Ab-positive MG (level IV evidence).^40,41^ In a prospective open-label study of 22 patients with refractory MG treated with rituximab, the median time to relapse was 17 months. In all, 14 patients taking additional prednisone were able to reduce the median daily prednisone dose from 25 to 7 mg after rituximab treatment (median follow-up of 29 months).^42^

Based on these data, two prospective clinical trials were initiated to further investigate the effect of therapeutic B-cell depletion. In the completed phase II BeatMG trial, 52 patients with an AChR-Ab-positive gMG (1:1) were randomized to a rituximab or a placebo treatment arm.^43^ The majority of patients initially took steroids alone, with one-third taking combination therapy consisting of prednisone plus one other immunotherapeutic agent. According to preliminary data, the study’s primary end point – defined as at least a 75% reduction in mean steroid dose between weeks 4 and 52 after rituximab treatment – was not met (60% reduction with rituximab *versus* 56% reduction with placebo therapy [one-sided odds ratio (OR): 1.14, 90% CI: 0–2.41]. The unexpectedly high steroid reduction in the placebo group and the only two rituximab doses administered were discussed as problematic.^44^ The study was underpowered and the primary end point may not have been very sensitive.^45^ The recently completed randomized-controlled phase III RINOMAX trial demonstrated that patients with new-onset AChR-Ab-positive gMG on a low-dose rituximab regimen (one infusion of 500 mg) were significantly more likely to meet the primary end point of minimal disease manifestation at 16 weeks than placebo-treated patients.^46^

Rituximab is well tolerated by most patients. Infusion reactions and severe, sometimes life-threatening anaphylactic reactions may occur and premedication is necessary. It should therefore only be used at a center with appropriate experience. For longer-term (>6 months) combination therapies (e.g. rituximab + another immunotherapeutic and/or + steroids), pneumocystis pneumonia prophylaxis (e.g. with cotrimoxazole 3×/week) should be considered. In view of the tolerability and the risks mentioned above, the indication for the continuation of therapy with rituximab should be carefully re-evaluated within the framework of regular control examinations, and possibilities for extending the therapy intervals should be exploited.

#### Complement inhibitors

*Eculizumab*. Eculizumab is a humanized monoclonal IgG-Ab that binds to the protein C5 of the complement system and is designed to prevent the formation of the membrane attack complex induced by C5b and thus damage to the neuromuscular endplate. Based on the data available to date in relation to potential complications with sometimes life-threatening infections, and in view of the lack of comparative and long-term data, and from an economic point of view, eculizumab should only be considered for the treatment of selected patients with refractory AChR-Ab-positive gMG. The main basis for approval is the results of an early phase II study and the REGAIN study.^4,47^ In the multicenter, randomized, double-blind, placebo-controlled phase III REGAIN study, 62 patients were treated with eculizumab as an add-on to ongoing baseline therapy, and 63 patients were treated with a placebo. The primary study end point (improvement in activities of daily living measured by the so-called worst rank ANCOVA analysis) was not met; however, almost all secondary end points (activities of daily living, quality of life, and muscle strength scales measured by ‘classical’ statistical comparison tests) showed a significant benefit of eculizumab, starting in the first 4 weeks of treatment and sustained over 6 months. In addition, compared with those on placebo, patients in the eculizumab group were significantly less likely to show clinical worsening and less likely to require ‘rescue’ therapy with PE or IVIg. In the open-label extension study of REGAIN, 117 patients were treated with 1200 mg of eculizumab every 2 weeks for up to 3 years (median: 23 months). The extension-phase exacerbation rate was 75% lower than the 1-year baseline rate before study entry (25 *versus* 102 events per 100 patient-years).^48^ A retrospective observational study shows that the use of eculizumab (20 patients) is safe and potentially superior to rituximab (57 patients) in terms of efficacy (change in QMG score at 12 months) in refractory patients.^49^

Eculizumab is administered as an intravenous infusion started at least 2 weeks after meningococcal vaccination. If vaccine efficacy cannot be waited for or vaccine success cannot be assumed due to immunotherapy, prophylactic antibiotic therapy should be started in addition to vaccination. Since quinolones can worsen MG,^50,51^ the following may be considered: rifampicin 600 mg p.o. every 12 h, taking into account interactions, or alternatively ceftriaxone 2 g i.v. every 24 h in case of intolerance. The first four administrations of eculizumab are at a dose of 900 mg once weekly. This is followed by administration of 1200 mg of eculizumab every 2 weeks starting at week 5. In children and adolescents weighing less than 40 kg, the dose must be adjusted accordingly. Therapy success can be assumed if the MG-ADL score is reduced by at least three points and the QMG by ⩾2 points. It is not yet certain how long eculizumab treatment should be continued in responders. In principle, there are no immunobiological arguments or safety concerns from previous long-term use of eculizumab that argue against continued therapy. Interestingly, late improvements in MG symptoms seem to occur in some patients with prolonged therapy, possibly reflecting later-onset repair mechanisms at the neuromuscular synapse. The extent to which it is possible to extend the intervals between infusions or even to pause therapy when the patient is stabilized is not currently supported by data. If no clinical improvement is observed after a 3-month treatment trial, discontinuation of therapy should be considered.^4,48^ However, available long-term data from the REGAIN cohort suggest that the proportion of responders increases from approximately 67% (<12 weeks) to 85% (⩾12 weeks).^52^

Eculizumab is associated with a 1000- to 2000-fold increased risk of meningococcal infection due to its mechanism of action.^53^ Therefore, in addition to vaccination, patients should carry an eculizumab patient card and immediately contact their treating physician or an emergency department and take an antibiotic (such as 600 mg rifampicin p.o., ciprofloxacin p.o.) if symptoms of meningococcal infection occur. In the REGAIN extension study, 19% of patients developed an infection of ‘special interest’ over 2 years including five cases with sepsis or septic shock and one case each with aspergillus, cytomegalovirus, and pseudomonas infection.^48^ Furthermore, disseminated gonococcal infections may occur.^54^ Follow-up for at least 3 months should also be performed after completion of treatment.

In the studies to date, eculizumab is used in addition to continuing immunotherapy. This add-on therapy makes sense from a pathogenetic point of view, as eculizumab does not affect the underlying autoimmune mechanism (i.e. production of auto-Ab) itself. Which drugs can best be combined with eculizumab currently remains a case-by-case decision, as no robust data are available.

*Ravulizumab*. A further development of eculizumab is the complement factor 5 inhibitor ravulizumab, in which the infusion frequency of maintenance therapy is 8 weeks instead of two. In the phase III CHAMPION trial, 26 weeks after initiation of therapy, the primary end point (improvement in MG-ADL from baseline) was significantly better in the ravulizumab group than in the placebo group. Data from the open-label extension phase of the CHAMPION study confirm the safety and efficacy of ravulizumab.^55^ Ravulizumab was approved by the European Commission on 23 September 2022.^5^ Infusion therapy is body weight-adapted (40–60 kg, 60–100 kg, >100 kg) on day 1 as well as day 15 at doses of 2400, 2700, or 3000 mg and every 8 weeks thereafter at doses of 3000, 3300, or 3600 mg. Safety precautions related to meningococcal vaccination and therapy, if necessary, are the same as those for eculizumab.

Another complement factor 5 inhibitor is zilucoplan, which was successfully tested in a phase III RAISE trial in the indication MG.^56^

#### Inhibitor of the FcRn: Efgartigimod

Efgartigimod is a human IgG1-Ab fragment designed to prevent the binding of (pathogenic) Ab to the so-called FcRn, which is important for IgG recycling. Efgartigimod can lower the concentration of auto-Ab directed against AChR and reduce disease activity (phase III study ADAPT,^57^ extension study: ADAPT-Plus; phase I and II^58,59^). In the multicenter, randomized, double-blind, placebo-controlled phase III ADAPT study, the primary study end point was met. Efgartigimod was administered intravenously in four doses per cycle at weekly intervals. The primary end point was clinically significant improvement (of ⩾2 points in the MG-ADL score for a duration of at least 4 weeks after the last infusion). Significantly, more patients with AChR-Ab-positive myasthenia improved on efgartigimod than on placebo (MG-ADL score; 67.7% *versus* 29.7%). Efgartigimod received approval in the United States on 18 December 2021. Approval in the EU followed on 10 August 2022.^7^ Approval is still pending in Switzerland.

Rozanolixizumab, another FcRn modulator, has been successfully tested very recently in the phase III MycarinG trial.^60^

### Therapy of crisis-like deteriorations, exacerbations, and crises (2.4.3)

**Table:** 

**Recommendation 2.4.3a**
In the event of an impending and manifest myasthenic crisis, rapid admission and treatment *must* be provided in a monitoring or intensive care unit with experience in neuromuscular disease. IVIG or PE/IA *must* be used in this situation.
Strong agreement

In the treatment of myasthenic exacerbations, PE and IVIG are considered equivalent.^19,61,62^ A comparative study of 84 patients with moderate to severe MG (QMG > 10.5) and clinical exacerbation showed comparable efficacy of IVIG and PE with respect to the primary end point of reduction in QMG score (69% for IVIG and 65% for PE) and duration of the crisis, as well as secondary clinical and electrophysiological end points over a 60-day observation period.^63^ Therefore, the decision for PE *versus* IVIG treatment will depend on individual patient factors such as concomitant diseases, in addition to local availability. For example, PE is contraindicated in sepsis and IVIG in hypercoagulability, renal failure, or existing hypersensitivity to Ig.^19^ The use of IVIG as a ‘bridging’ therapy (e.g. in patients with poorly controlled diabetes or in patients in whom steroids cannot be successfully reduced) is preferred by some experts over monthly PE for practical reasons.^19^

#### Intravenous immunoglobulin

*Short-term treatment*: IVIG should be administered at 0.4 g/kg bw for five consecutive days,^64^ alternatively 1 g/kg bw for 2 days.^65–67^ In individual cases, a lower total dose of 1 g/kg bw may be sufficient. IVIG shortened the time of ventilatory requirement in myasthenic crisis.

Likewise, IVIG may be useful for stabilizing unstable conditions before surgery (including thymectomy) or before starting high-dose steroid therapy for severe myasthenia.

*Maintenance therapy*: No data are available from randomized clinical trials on the clinical value of IVIG as maintenance therapy – either alone or as an add-on therapy to existing immunotherapy medication. Based on expert knowledge, IVIG can be used for maintenance therapy over time in individual cases (initially 5 g/kg × 0.4 g/kg bw as a pulse, then 1 g/kg × 0.4 g/kg bw every 4–8 weeks or higher doses if clinically necessary) outside the indication for acute exacerbation or myasthenic crisis.^68–72^ Two controlled trials are evaluating the efficacy of IVIG in chronic myasthenia treatment and as steroid-sparing therapy over a 12-month period (phase II: NCT02473952, NCT02473965). The studies have been completed and the results can be viewed in the registry entry. A publication with a detailed analysis of the data is not available. The multicenter, prospective, albeit open-label and uncontrolled phase III GTI1305 trial argues for the safety of IVIG-C (2 g/kg bw) in the treatment of patients with severe acute exacerbations of MG or myasthenic crisis.^73^ Its use as a continuous therapy in MG patients who have a (relative) contraindication to classical immunotherapy either because of comorbidity (e.g. preexisting severe osteoporosis, recurrent infections with resistant germs, sepsis, advanced age), pregnancy, or multiple intolerance reactions seems worth considering.

*Subcutaneous Ig (SCIG)*: SCIG has the potential advantage of more consistent serum levels compared to IVIG, resulting in reduced wear-off effects at the end of the treatment cycle. A prospective, open-label phase III study in patients with mild to moderately severe myasthenic exacerbation (increase in MGFA from I to II or III, or from II to III) demonstrated that weekly SCIG at a dose equivalent to IVIG administration could improve MG scores.^74,75^ The treatment could be an alternative for patients with comorbidities or poor venous conditions. Whether the efficacy of SCIG and IVIG is comparable in maintaining remission has not yet been investigated.

#### Plasmapheresis

*Short-term treatment*: PE unselectively removes non-corpuscular blood components *via* blood centrifuges or plasma separators with vascular access *via* large-volume peripheral or central venous catheters. The indication is a myasthenic crisis. In addition, PE can be used in other refractory courses to stabilize unstable conditions prior to surgery (including thymectomy) or prior to initiation of high-dose steroid therapy for severe myasthenia. Typically, 5–10 treatments are given (initially daily; usually one to one and a half times plasma volume every other day) until clinical stabilization is achieved. Without concomitant immunotherapy, the clinical effect is limited to a few weeks.^76,77^ Substitution with human albumin (or albumin PP (polypropylene) for jMG) is often but not always done after each treatment. In secondary Ab deficiency syndrome (IgG < 150 mg/dl), substitution with polyvalent IgG is recommended. Transient depletion of coagulation factors limits replacement frequency and must be considered when anticoagulation is indicated elsewhere. Multimorbid, elderly patients, especially those with cardiac disease, are at risk for volume loading.

*Maintenance therapy*: Study results on the influence of PE *versus* immunotherapy on the long-term course of myasthenia are lacking.^78^

#### Immunoadsorption

IA is now widely performed instead of classical PE and is considered equally effective in myasthenia.^79,80^ Comparable effectiveness in the treatment of myasthenic crisis was demonstrated in a randomized-controlled trial.^81^ The logistic and technical requirements correspond to those of PE. In this procedure, either IgG is removed semi-selectively with a tryptophan-polyvinyl gel matrix^77^ or cost-intensive protein A-columns,^62^ or IgG of subclasses IgG 1, 2, and 4 are eliminated selectively by binding to protein A-sepharose.^82^ Advantages of IA include the lack of need for substitution of plasma proteins by the administration of fresh frozen plasma or human albumin, little disruption of coagulation ratios, and the possibility of much higher exchange volumes without critical volume fluctuations and reduced circulatory burden. Compared with PE, IA has decisive advantages in pregnancy and circulatory unstable patients; in addition, the duration of treatment is usually shorter than with PE.

### Antibody-specific therapy features (2.4.4)

**Table:** 

**Recommendation 2.4.4a**
For MuSK-Ab-positive myasthenia with mild/moderate activity, symptomatic therapy with AChE-I *must* be complemented by GKS ± AZA as first-choice therapy for disease course modification. Rituximab^1^ *must* be used for patients with (highly) active course (including refractory to therapy). Second-choice therapies *must* be selected analogous to AChR-Ab-positive MG, while FcRn modulators (efgartigimod*) *may also be considered*. *Rituximab and efgartigimod are *off-label* in these indications.
Strong agreement

In addition to the MGFA classification and the current disease activity [mild/moderate MG *versus* (highly) active MG], the auto-Ab findings are increasingly decisive for the therapeutic approach. For AChR-Ab-positive MG, the data situation is best due to the relative prevalence within MG. The use of eculizumab is approved (only) for AChR-Ab-positive MG. The efficacy of thymectomy in terms of the likelihood of achieving disease remission or saving immunotherapy is also best demonstrated here. For MuSK-Ab-positive MG, in particular, therapy with eculizumab is not approved and also immunopathogenetically not reasonable due to the IgG4 antibody subtype. Here, therapy with rituximab has become the established choice. For LRP4-Ab-positive MG, the benefit of thymectomy is currently unclear. Therapy with eculizumab is not approved, but it is immunopathobiologically useful. Forms of myasthenia currently classified as seronegative are treated like AChR-Ab-positive MG. There is no approval for therapy with eculizumab.

Beyond AChR-Ab-positive MG, most are known about MuSK-Ab-positive MG. This is pathogenetically distinct from AChR-Ab-positive MG. It is predominantly mediated by auto-Ab of the non-complement-binding IgG4 subclass,^83,84^ so C5-blocking drugs (e.g. eculizumab, ravulizumab, zilucoplan) have no target here. Patients with MuSK-Ab-positive MG may respond poorly to AChE-I and more often tolerate it poorly.^85,86^ They also appear to respond better to PE/IA than to IVIg, although comparative studies are lacking due to the rarity of the disease. Case series suggest that B-cell-depleting treatment with rituximab is effective, particularly in MuSK myasthenia, and should therefore be considered early as a first-choice treatment option as well.^19^ In MuSK myasthenia rituximab might induce long-term remission in some patients without the need for further doses. Thymomas and other thymic pathologies very rarely occur associated with MuSK myasthenia. Thymectomy is therefore not indicated for patients with MuSK myasthenia according to current data, nor is treatment with complement inhibitors.

### Surgical therapy (2.5)

### Thymectomy (2.5.1)

**Table:** 

**Recommendation 2.5.1a**
(a) In patients with AChR-Ab-positive gMG between the ages of 18 and 65 years, thymectomy (transsternal or minimally invasive) *should* be performed as early as possible within 2 years and no later than 5 years after the diagnosis is confirmed. (b) Thymectomy *may also be considered* in seronegative gMG and LRP4-Ab-positive gMG with high disease activity during the first 2 years of disease, if possible. (c) MuSK-Ab-positive gMG patients *must not* undergo thymectomy. (d) Thymectomy *may also be considered* in generalized AChR-Ab-positive jMG and decided on an individual basis. (e) In children and adolescents aged 5–12 years, thymectomy *should* be performed only after failure of standard drug therapy (AChE-I, GKS). In adolescents aged 13 years and older, the procedure *should* be continued as in (a).
Strong agreement

The high value of thymectomy for the treatment of MG is supported by the results of the prospective randomized MGTX study. The MGTX study included patients with AChR-Ab-positive generalized myasthenia who were 18–65 years of age and whose disease had lasted not longer than 5 years. There was a significant benefit for the thymectomized patients in terms of myasthenic symptoms, accompanied by a steroid- and immunotherapy-sparing effect that occurs approximately 1 year after thymectomy and persists at least for 5 years.^2,3,87^ Thus, compared with those treated with prednisolone alone, patients who underwent thymectomy had a significantly lower QMG score 3 years after study entry (6.1 *versus* 9.0; *p* < 0.001), a lower mean requirement for prednisone (every 2 days 44 mg *versus* 60 mg, *p* < 0.001) with less frequent use of long-term immunosuppression by AZA (17% *versus* 48%, *p* < 0.001).^3^ Thymectomy is therefore considered the standard of care for the above-mentioned patient group (class 1 evidence).

Thymectomy should usually be performed as elective surgery in clinically stable patients. If improvement is necessary preoperatively, the options of therapy with GKS or, as crisis intervention, IVIg or PE or IA are available.

The benefit of surgical therapy for MG has been demonstrated by class 1 evidence only for the above-mentioned subgroup and the surgical procedure of median sternotomy. However, since then, similar results of functional improvement of MG after thymectomy by minimally invasive surgical techniques have been observed. In one study, although uncontrolled in design, an astonishing improvement of up to 80% cumulative complete stable remission (CSR) was observed.^88^ Due to technical innovation, the robot-assisted technique of thoracoscopic thymectomy may offer a comparatively gentle alternative for the various anatomically challenging scenarios (much tissue in the anterior mediastinum, high BMI, young children, anatomical aberrations). However, in case of an emergency, the need for conversion to thoracotomy or even toward sternotomy must be considered.

Additional data are needed to assess the role of thymectomy for subgroups not included in the MGTX study. The decision to perform a thymectomy should be individualized and multidisciplinary in a specialized myasthenia center. Older patient surgery is an option based on new evidence. In ocular onset MG, thymectomy may help to prevent generalization. In addition, thymectomy may positively affect the CSR parameter. This is more likely if thymectomy is given when symptoms are purely ocular rather than after generalization.^89,90^

In seronegative MG (for Ab against AChR, MuSK, and LRP4), after exhaustion of all non-surgical measures, the indication for thymectomy may be justified in individual cases from a neurological point of view in an individual therapy concept. For the subgroup of MuSK-Ab-positive MG, no indication for thymectomy is currently seen.^91^

### Thymectomy in case of suspected thymoma (2.5.2)

**Table:** 

**Recommendation 2.5.2a**
(a) Every MG patient *must* be evaluated for the presence of a thymoma. Thymomas *must* be surgically removed at any age regardless of the severity of myasthenia. In exceptional cases, depending on the imaging findings, complex treatment by neoadjuvant chemotherapy or radiochemotherapy *should* be performed. Depending on the histopathological findings, adjuvant postoperative radiotherapy *should* be performed. b) If the patient is not suitable for surgery, and thymoma is suspected, a biopsy and, if necessary, conservative therapy (usually radiotherapy) *must* be performed. c) Depending on the preoperative staging and the experience of the surgeon, minimally invasive surgical techniques *may be considered* in addition to transsternal surgical techniques. d) Even in children and adolescents, a thymoma – despite its rarity – *must* be excluded on the basis of image morphology.
Strong agreement

Thymomas and thymic carcinomas are classified as malignant and predominantly show locoregional infiltrative growth into the directly surrounding adjacent anatomic structures.^92^ When a thymoma is detected, there is an indication for surgery regardless of the extent of MG. In any case, after histologic workup of the resectate, the histologic thymoma type according to the WHO classification,^93^ tumor stage according to both the Masaoka-Koga classification^94^ and the tumor-node metastasis classification,^95^ and resection status (R0, R1, and R2) should be reported in the findings. Obtaining a reference pathology should be considered.

Surgical therapy of tumors of the thymus should always be stage-adapted with the goal of complete resection. Thus, radical thymectomy including thymomectomy should be performed as standard. In case of infiltration of adjacent organs (e.g. lungs, vessels) in stage III, en bloc resection of these structures should also be performed, and depending on the extent of tumor infiltration, surgical resection should be performed primarily or after induction therapy.^96,97^ Randomized-controlled data regarding neoadjuvant or adjuvant therapy do not exist to date. A consensus decision should be reached by an interprofessional team of thoracic surgeons, oncologists, and radiation therapists.

Elderly and multimorbid patients can be treated with palliative radiotherapy if there is a small tumor spread, slow progression of the thymoma, and well-compensated myasthenic symptoms. Primarily, inoperable thymomas and recurrences can be treated neoadjuvant with Somatostatin^®^ plus GKS if the octreoscan is positive.^98,99^

### Therapy of individual symptoms (2.6)

#### Fatigue syndrome

The prevalence of fatigue syndrome in MG is increased compared to the normal population and varies between 44% and 82%, with women being affected more often than men.^66,69^ The prevalence correlates with the severity of myasthenic syndrome, but approximately one-third of MG patients in (pharmacological) remission also have fatigue syndrome.^70^ The presence of fatigue syndrome is associated with a lower quality of life and depressive symptoms (only cross-sectional studies are available; therefore, no statement on causality is possible).^69^ Data on fatigue treatment in MG patients are limited, partly because fatigue was not included as an outcome parameter in previous intervention studies. Recent data increasingly suggest that consistent myasthenia-specific treatment can also positively influence fatigue syndrome. In the REGAIN study, there was a significant improvement in fatigue syndrome in the eculizumab group compared to the placebo group.^71^ Similar effects have been shown for corticosteroids, IVIg, and PE treatments, although only uncontrolled studies are available for these.^72,73^ If the effect of myasthenia-specific medication is insufficient, a therapeutic trial with selective serotonin-norepinephrine reuptake inhibitors, tricyclic antidepressants, or selective serotonin reuptake inhibitors can be attempted^76^ However, it is of key importance to educate patients that fatigue syndrome is part of the clinical spectrum of MG and can occur independently of muscular fatigue. Patients should be encouraged to not overprotect themselves or exert themselves beyond the limits set by MG.^76^ Patients’ self-efficacy should be enhanced *via* modification of lifestyle factors, such as good sleep hygiene, balanced diet, avoidance of excessive alcohol consumption, and performance of aerobic exercise. If necessary, additional psychological support may be helpful.^77^

#### Therapy of ocular myasthenia

Ocular myasthenia is also treated symptomatically with pyridostigmine, although often a satisfactory effect cannot be achieved. Using steroids, a satisfactory effect is often attained with 0.5–1 mg/kg body weight after 2–4 weeks. Steroid-sparing use of long-term immunotherapeutics may also be necessary in ocular myasthenia. Thymectomy plays a secondary role in purely ocular symptomatology and the absence of thymoma evidence, as controlled study data are lacking. Thymectomy of oMG may be considered without signs of generalization if other MGTX criteria are present, especially if there is evidence of thymic hyperplasia on chest imaging.

To correct ptosis, in addition to mechanical aids (eyelid retractor on glasses, clear tape), permanent eyelid retraction can produce excellent results in courses refractory to medical therapy. Persistent double vision should initially be treated transitionally by an alternate covering of one eye. In the absence of remission under medical therapy, the ocular malposition often stabilizes, which can be treated by prism correction (prism foils for spectacle lenses) in the case of small malposition angles, or squint correction surgery in the case of larger malposition angles.

### Therapy of jMG (2.7)

**Table:** 

**Recommendation 2.7a**
Juvenile gMG and oMG *must* be treated with drugs in the same way as adult MG – taking into account Ab subtype and disease activity. In prepubertal children, spontaneous remissions should be considered. Therapy with rituximab and eculizumab in juvenile gMG *should* also be discussed individually as an off-label option. In crisis, PE/IA *can be considered*, and the administration of IVIg *can be considered*.
Strong agreement

In children and adolescents with jMG, the specificities of age and disease course must be considered in therapeutic decisions. This concerns higher remission rates (15–39%) and frequently isolated ocular forms in prepubertal children (26–38%).^15^ In children and adolescents with jMG, the evidence regarding immunotherapy and thymectomy is limited and based solely on retrospective studies and clinical experience. The recommendation was summarized in a recent international workshop.

#### Thymectomy

Retrospective data show that even in patients with jMG, thymectomy reduces the required dose of immunotherapeutic agents or allows their long-term omission. The indication for thymectomy is made according to the impairment of the child’s development under exhaustion of drug therapy (e.g. growth retardation). Children under 5 years of age can in theory also be operated on successfully using minimally invasive techniques; interdisciplinary individual case decisions are required in this context. Data on the immunologic consequences of thymectomy in the early years of life come mainly from cardiac surgery for congenital heart diseases. Early thymectomy may be associated with a reduction in T-cell subpopulations and TCR diversity even in the long term.^100^ However, clinically relevant late effects on the immune system have not been observed, especially in thymectomized jMG patients.^101^

#### Symptomatic therapy

In children and adolescents, the dose of pyridostigmine is calculated as 1–7 mg/kg bw/day, the single dose at the beginning as 0.5–1 mg/kg bw; a maximum daily dose of 450 mg should not be exceeded.

#### Immunotherapy

In jMG, attention should be paid to the long-term effects of prolonged steroid therapy on growth velocity and bone density. Therefore, after reaching the initial recommended dose (0.5–1.5 mg/kg bw), a reduction of this dose and the lowest possible long-term dose or discontinuation should be aimed.

Steroid-sparing therapy with AZA is recommended in generalized jMG when steroid therapy fails to achieve sufficient effect or cannot be continued due to steroid dependence or side effects. In case of contraindication or side effects under AZA, the use of MMF is reasonable. There are also positive results for the use of tacrolimus for refractory jMG.

Therapy with rituximab (375 mg/m^2^ body surface) has been reported in a few patients with jMG, predominantly with positive effects. Data on the use and effect of eculizumab therapy in children are not available; a study is currently underway. MTX and CSA are not used.

In jMG, IVIg can also be used as maintenance therapy every 4–6 weeks if stabilization cannot be achieved by the previous immunotherapy.

### Vaccinations with MG (2.8)

**Table:** 

**Recommendation 2.8a**
Vaccinations including COVID-19 vaccinations *must* be performed in MG including jMG as usual according to Robert-Koch-Institute recommendations.^i^ This applies in particular to live vaccinations and the indication for vaccination in jMG. Prior to therapy with complement inhibitors, vaccination against meningococci of serogroups A, C, Y, W135 (1 × Menveo^®^), and B (2 × Bexsero^®^ at intervals of 4 weeks) *must* be performed.^ii^ Live vaccinations *should not* be performed under immunotherapy. If time permits, required vaccinations *should* be completed at least 4 weeks prior to initiation of immunotherapy. In the case of rituximab (RTX) therapy, vaccinations *must* be given 1 month prior to a scheduled dose, or at least 1 month after RTX therapy,^iii^ or prior to the next dose. ^i^The competent authority for Switzerland is the Federal Office of Public Health. ^ii^If vaccination until the start of therapy is not possible or if it is not possible at all, antibiotic prophylaxis must be given until 2 weeks after vaccination or for the entire duration of therapy. ^iii^Optimal would be a period of 4–6 months. Shorter periods are possible, but it must be assumed that the humoral immune response is then impaired.
Strong agreement

Precise recommendations for the time interval between rituximab administration and vaccination are not possible. No sufficient data are available for MG. In general, the longer the interval between rituximab administration and vaccination, the greater the humoral vaccination success. In general, 6 months is considered sufficient, a period in which CD20-positive B cells have recovered in most patients. Data from patients with multiple sclerosis during the COVID-19 pandemics confirm this.^102^ However, it must be kept in mind that the cellular vaccine response, which plays an essential role in pathogen defense and disease progression, is not or only marginally affected by rituximab. Therefore, from a risk–benefit perspective, it must be decided whether earlier timing of vaccination after rituximab administration might be more beneficial.

Vaccinations should be given according to the national vaccination recommendations.^103,104^ In Germany, the STIKO (Ständige Impfkommission; Permanent Vaccination Commission) of the Robert-Koch-Institute (part of the German Federal Ministry of Health) recommends that vaccinations should be refreshed, if possible, in all patients prior to immunotherapy. Immunotherapy-treated patients in particular should have especially good vaccination protection. There are no data to suggest that the general recommendations made by the STIKO applicable to immunosuppressed patients should be modified for immunosuppressant-treated patients with myasthenia. In principle, inactivated vaccines can be used under immunotherapy without a risk of more frequent or severe side effects, but vaccination success may be reduced or absent. Therefore, if possible, vaccinations should be given before immunotherapy is started (vaccinations to be completed at least two, preferably 4 weeks prior to treatment), or at a time when immunotherapy has as little effect as possible. Live vaccinations (e.g. varicella, two doses 4 weeks apart) are contraindicated during immunotherapy and, if indicated, should therefore be completed 4–6 weeks before starting therapy, if possible. Yellow fever vaccination (live vaccine, relatively high replication rate of vaccine virus) is also contraindicated after thymectomy.

Meningococcal vaccination prior to complement inhibitor therapy is a special situation. At least 2 weeks prior to the first administration of complement inhibitors, vaccination against meningococcal serogroups A, C, Y, W135 (1 × Menveo^®^), and B (2 × Bexsero^®^ 4 weeks apart) must have been given. If the effectiveness of the vaccination cannot be waited for or if a vaccination success cannot be assumed due to the immunotherapy, prophylactic antibiotic therapy should be started in addition to the vaccination.

Due to B-cell depletion under rituximab with subsequent confirmed impaired immune response to different vaccines, consideration should be given to vaccination against meningococcal serogroups A, C, Y, W135, and B also before a planned rituximab treatment. Although this is not mandatory, it allows subsequent conversion to targeted complement inhibition (e.g. with eculizumab) in the event of an inadequate therapeutic response to rituximab.

Currently, available data indicate an increased risk for a severe course in the case of COVID-19 infection in MG patients.^105^ Therefore, COVID-19 vaccination is recommended for MG patients, especially in the presence of other myasthenia-specific risk factors such as bulbar and/or respiratory symptomatology and/or rituximab therapy.^106^ The currently available mRNA and vector vaccines are not live vaccines. Therefore, no specific safety concerns arise for use in myasthenic patients.

Patients under 18 years of age must also be vaccinated against *Haemophilus influenzae*.

### Special aspects of the management of myasthenia patients in the context of family planning (2.9)

**Table:** 

**Recommendation 2.9a**
If the patient wishes to have a child and pregnancy has occurred, specific counseling *should* be provided at a center specializing in myasthenia.
Strong agreement

**Table:** 

**Recommendation 2.9b**
Because of the risk of neonatal MG and peripartum deterioration of maternal MG, child delivery *should* be planned in a maximum care hospital, including those with neonatology and neuropediatric expertise and intensive care monitoring capabilities.
Agreement

### Medications that may worsen MG (2.10)

In MG, additional administration of medications may be necessary due to other diseases or medical conditions. It is important to keep the following in mind: Some medications may worsen symptoms of MG or cause MG to erupt. While for some of the drugs, this suspicion is considered scientifically confirmed, for the majority of drugs this currently remains scientifically unconfirmed. Moreover, the decision to treat with these drugs is based not only on the MG but also on the justifying indication. It is important to consider whether there are any effective alternative drugs available at all. The most important substances and substance groups are listed in Supplemental Table 4.

### Congenital myasthenic syndromes (2.11)

**Table:** 

**Recommendation 2.11a**
If CMS is suspected, molecular genetic diagnostics *must* be performed to confirm the diagnosis and plan therapy.
Strong agreement

**Table:** 

**Recommendation 2.11b**
In Ab-negative myasthenia, depending on the response to immunotherapy, the differential diagnosis of CMS *should* be considered and genetic diagnosis should be performed if necessary.
Strong agreement

CMS are a genetically and phenotypically heterogeneous group of disorders caused by faulty or impaired neuromuscular transmission and characterized by the leading symptoms of muscle weakness and abnormal exercise intolerance (Supplemental Table 5). The severity can vary enormously and depends on the underlying genetic cause; it ranges from mild impairment to life-threatening situations in the neonatal period or the context of crisis-like deterioration in older children and adolescents. Less commonly, initial manifestations in later adulthood are also possible.

Abnormal muscle fatigue may affect various muscle groups: limb-girdle, distal, proximal, cervical/axial, respiratory, ocular, facial, and bulbar muscles. Smooth muscles and cardiac muscles are generally not affected.

Symptoms usually manifest at birth or in the first 2 years of life. Nevertheless, initial manifestations have been described into old age, although much less frequently; they are then often misdiagnosed as seronegative MG (especially mutations in the *RAPSN*, *DOK7*, *GMPPB* genes, and slow-channel CMS).^107–114^

CMS are rare overall, with an estimated prevalence of 1–9/10^6^. No data are available for the subtypes of CMS.^114^

## Diagnosis and therapy of LEMS (3)

**Table:** 

**Recommendation 3a**
(a) In LEMS, symptomatic therapy *must* be with amifampridine or 3,4-DAP and pyridostigmine. For pLEMS, tumor treatment *must* be performed as usual. GKS *must* be used for the treatment of LEMS as a basic immunotherapeutic agent at a dosage appropriate to the severity of the disease for as short a period as possible, taking into account comorbidities, contraindications, and side effects. AZA *must* be used for steroid-sparing therapy. (b) As an alternative to AZA, treatment with MMF, CSA, tacrolimus, or RTX *should* be considered if appropriate. MTX *should* be avoided, especially in the case of pLEMS in BC and radiation of the lung. IVIg or, if appropriate, PE/IA *must* be used to treat the crisis worsening.
Strong agreement

### Disease definition and etiology

LEMS is a rare presynaptic neuromuscular disorder caused by auto-Ab against the P/Q type of VGCC of peripheral nerves. These Ab are pathognomonic and can be detected serologically in approximately 85% of patients with LEMS.^115^

LEMS can be idiopathic (iLEMS; mostly women younger than 50 years) or, less commonly – especially in small-cell lung cancer (SCLC) – as a paraneoplastic disease (pLEMS; mostly in long-time smokers).^116^ In rare childhood LEMS, an association with lymphoproliferative disorders and neuroblastoma is found.^117^ The antigenic stimulus of pLEMS originates from VGCC expressed by tumor tissue^118^; in iLEMS, the trigger for the emergence of auto-Ab is unknown. There are isolated case reports of a drug-induced immune process in LEMS, as in therapy with alemtuzumab or with a checkpoint inhibitor.^119,120^

Clinically, a triad consists of:

- Proximally emphasized load-dependent weakness (usually without involvement of the ocular and bulbar musculature)
- Hyporeflexia (areflexia), and
- Autonomic disturbances such as, among others, increased sweating and a dry mouth due to decreased salivation due to impaired cholinergic transmission.

The weakness typically spreads caudally (hip girdle) to cranially as it progresses. If the autonomic disturbances occur within the first year of illness, then this is indicative of a paraneoplastic genesis of LEMS.^121^

Clinical neurophysiology reveals the so-called Lambert triad, consisting of:

- Low sum action potentials in motor neurography; on hand muscles, frequent peak-to-peak amplitude below 2 mV.
- Pathological decrement (⩾10%) in low-frequency serial stimulation (2–3 Hz), typically showing no rebound in amplitudes after the fifth summation action potential.^122^
- Pathological increment (>60%). Maximal voluntary innervation over 10 s significantly increases the amplitude of the motor summing action potentials.^123^ This amplitude increase, the increment, is explained by an increased calcium influx during volitional activity. During prolonged muscle tension, the increment rapidly decreases. In LEMS without serological evidence of Ab against P/Q-type calcium channels, the electrophysiological abnormalities are often less pronounced, such that in seronegative patients, an increment of 60% should be considered diagnostic.^124^ An increment can also be visualized *via* high-frequency but very painful serial stimulation with stimulation frequencies above 20 Hz. This is a diagnostic option only in, for example, intensive care medicine in patients with suspected LEMS who cannot innervate sufficiently at will.

### Tumor search

pLEMS primarily affects long-time smokers, although the disease may also occur many years after abstinence. Clinically, it is characterized by rapid progression, with autonomic symptoms occurring within the first year of the disease.^121^ The *Dutch-English LEMS Tumor Association Prediction* (DELTA-P) score was developed as a clinical score to allow the prediction of paraneoplastic genesis in LEMS.^125^ The following criteria are assessed with one point each at baseline or within 3 months of disease onset:

1. *D*ysarthria, dysphagia, neck muscle weakness – bulbar participation
2. *E*rectile dysfunction – erectile dysfunction in men
3. *L*oss of weight – weight loss ⩾5%
4. *T*obacco at onset – smoking at the time of illness
5. *A*ge – age ⩾50 Jahre
6. *P*erformance in Karnofsky score 0–60 – Karnofsky index < 70

With a score of 3 or more points, pLEMS was present in >90%.

Anti-SOX1-Ab are found in 65% of patients with pLEMS, whereas they are found in only 5% of patients with iLEMS. Therefore, they may be additionally helpful in assessing the etiogenesis of LEMS.^126,127^

Most SCLC in patients with LEMS are detected within 2 years of the onset of neurologic symptoms (usually at a limited stage).^128,129^ Therefore, all patients with LEMS, regardless of their individual risk for SCLC, should receive a chest CT and, if the findings are unremarkable, a ^18^F-fluorodeoxyglucose positron emission tomography/CT.^128,129^ Due to its low sensitivity, a chest X-ray is not suitable for tumor detection. In case of unremarkable findings, screening should be performed every 3–6 months for at least 2 years, depending on the risk constellation. Such screenings detect 91% of all SCLC within 3 months and 96% of all SCLC within 1 year.^128,129^ Subsequently, screening based on individual risk assessment every 12 months for up to three additional years is reasonable.^130^

### Therapy

As in MG, drug therapy is based on a symptomatic and an immunotherapeutic approach. For symptomatic treatment of LEMS, amifampridine (3,4-DAP), based on four small placebo-controlled trials with a total of 44 patients, is available,^131–135^ which in its further development to amifampridine phosphate (3,4-DAPP) has been approved for LEMS in orphan drug status in Europe since 2010. The efficacy of amifampridine phosphate was confirmed by a pivotal phase III study.^136^ The recommended starting dose of amifampridine is 15 mg/day and can be increased by 5 mg every 4–5 days to a maximum daily dose usually of 60 mg. Amifampridine is usually divided into three to four doses daily with meals; a single dose should not exceed 20 mg (Supplemental Table 6).

Based on expert knowledge, pyridostigmine may be given in combination with amifampridine.^137^ Pyridostigmine also increases saliva production, which may help improve the dry mouth that is often very bothersome in LEMS.

In the case of an inadequate response to amifampridine, immunotherapeutics should be used. As with MG, based on expert knowledge, the recommendation is to initially start a combination therapy of GKS and AZA.^62,138,139^ The specific recommendations for dosing and therapy monitoring are analogous to MG. Formally, there is positive evidence for the use of IVIg in LEMS.^67,135^ IVIg should be recommended as helpful in the treatment of LEMS on this basis and on the basis of expert evidence, both as short-term and long-term therapy.^68,135^ For therapy with PE and IA, there are only single case reports and small case series. Few single case reports and expert knowledge on successful treatments with MMF, CSA, rituximab, and cyclophosphamide (outside of tumor treatment) may justify their use in the context of individual curative trials in experienced centers.

Drugs that may worsen MG may also have an unfavorable impact on LEMS.

### Tumor therapy in LEMS

Therapy of pLEMS focuses primarily on effective treatment of the tumor by means of (neo-)adjuvant chemotherapy, radiation, and, if necessary, surgical removal. An initiated and effective symptomatic treatment with amifampridine and, if necessary, pyridostigmine should be continued. Steroids may be used based on expert knowledge in pLEMS; the use of immunotherapeutics is based on clinical symptoms after tumor therapy and long-term prognosis and may be omitted during chemotherapy. The presence of LEMS is a favorable prognostic factor in terms of survival in SCLC.^140,141^ Experience has shown that even with successful tumor therapy, the LEMS persists.^142^

## Care coordination in myasthenic syndromes (4)

**Table:** 

**Recommendation 4a**
Multimodal (pharmacologic and nonpharmacologic) therapy of MG including thymectomy *should* be done in or in close coordination with myasthenia centers.
Agreement

Diagnosis and therapy of MG and LEMS can be performed predominantly in the outpatient setting if the presentation is clear. Repeated inpatient care is required on an individual basis, as in the case of differential diagnostic difficulties. In case of impending crisis-like deterioration or a crisis, inpatient care with intensive care expertise and access to escalation strategies (administration of IVIg and/or apheresis procedures) is absolutely necessary. Patients with generalized courses should be treated at least once in an experienced myasthenia center (clinic or specialized outpatient facility). Childhood and juvenile forms of the disease should be cared for in a neuropediatric facility with experience in neuromuscular disease. In the transition to adulthood, these patients should be managed in a structured transition process. The need for inpatient hospitalization (German Appropriate Evaluation Protocol criteria) is usually given in severe courses, in case of impending crisis, and also for the initiation of antibody therapies.

## Final comment

The value of practice guidelines, especially in the area of neuromuscular diseases, depends on sufficient professional diversity of the guideline committee members, including not only knowledge of routine clinical issues of diagnosis and therapy but also on issues of epidemiology, pathobiology, biomarkers, and treatment economics, as well as the patient perspective.^143^ The German guideline largely takes this approach into account. For example, experts in thymic pathology and thoracic surgery were included for input on pathobiological and therapeutic issues of the thymus. Another special feature of our guideline is the consideration of CMS as an important differential diagnostic disease group of seronegative myasthenia, which was mainly elaborated on by neuropediatric and human genetic experts. The vital patient perspective was taken into account *via* a representative of the board of the German Patient Organization for Myasthenic Syndromes in the steering group of the guideline commission. The German Myasthenic Society represents more than 3500 myasthenic patients in Germany.

The possibilities for therapy of MG are currently changing rapidly. In addition to several already successfully completed phase III studies, a large number of further phase II and phase III studies are ongoing. This poses some challenges to clinical practice, for example, which patients should be treated with modern immunomodulators. The German guideline has taken a new approach by introducing disease activity as a decision criterion for the use of modern immunomodulators. However, currently, there are only clinical criteria to define disease activity. The development of evidence-based markers of disease activity, including biomarkers, was recommended by the guideline committee as a key research priority. Similarly, more real-world data will need to be considered in the future to better assess the incremental benefit of expensive new immunomodulators. Furthermore, it will also be necessary to integrate health economic expertise into guideline work. The German guideline aims to remain up to date. It is therefore designed as a living guideline to integrate the currently rapidly changing treatment options for myasthenia gravis.

## Supplemental Material

sj-docx-1-tan-10.1177_17562864231213240 – Supplemental material for Guideline for the management of myasthenic syndromes Click here for additional data file. Supplemental material, sj-docx-1-tan-10.1177_17562864231213240 for Guideline for the management of myasthenic syndromes by Heinz Wiendl, Angela Abicht, Andrew Chan, Adela Della Marina, Tim Hagenacker, Khosro Hekmat, Sarah Hoffmann, Hans-Stefan Hoffmann, Sebastian Jander, Christian Keller, Alexander Marx, Arthur Melms, Nico Melzer, Wolfgang Müller-Felber, Marc Pawlitzki, Jens-Carsten Rückert, Christiane Schneider-Gold, Benedikt Schoser, Bettina Schreiner, Michael Schroeter, Bettina Schubert, Jörn-Peter Sieb, Fritz Zimprich and Andreas Meisel in Therapeutic Advances in Neurological Disorders

## Declarations

**Ethics approval and consent to participate:** Not applicable.

**Consent for publication:** Not applicable.

**Availability of data and materials:** Not applicable.
