# Erratum to Guideline for the management of Myasthenic Syndromes. *Therapeutic Advances in Neurological Disorders*. Vol. 16(1): 1–31. DOI 10.1177/17562864231213240

**DOI:** 10.1177/17562864241246400

**Published:** 2024-04-30

Due to administrative error, the author’s name Ulrike Schara-Schmidt and her affiliation were mistakenly removed from author byline and affiliation section.

Hence, the revised list of authors and Ulrike Schara-Schmidt’s affiliation should be read as follows:

Heinz Wiendl, Angela Abicht, Andrew Chan, Adela Della Marina, Tim Hagenacker, Khosro Hekmat, Sarah Hoffmann, Hans-Stefan Hoffmann, Sebastian Jander, Christian Keller, Alexander Marx, Arthur Melms, Nico Melzer, Wolfgang Müller-Felber, Marc Pawlitzki, Jens-Carsten Rückert, Ulrike Schara-Schmidt, Christiane Schneider-Gold, Benedikt Schoser, Bettina Schreiner, Michael Schroeter, Bettina Schubert, Jörn-Peter Sieb, Fritz Zimprich and Andreas Meisel

**Ulrike Schara-Schmidt**

Neuropädiatrie, Klinik für Kinderheilkunde I, Universitätsklinikum Essen, Essen, Germany

The dose regime has been updated as follows in Section ‘Complement inhibitors:’

Infusion therapy is body weight adapted (40–60, 60–100, >100 kg) on day 1 at doses of 2400, 2700, or 3000 mg and on day 15 and every 8 weeks thereafter at doses of 3000, 3300, or 3600 mg.

Also the Ulrike Schara-Schmidt’s contribution details have been added as follows:

**Ulrike Schara-Schmidt:** Conceptualization; Resources; Writing – review & editing.

*The article has been updated to reflect the change.

