# Supplementary material for: Guideline for the management of myasthenic syndromes
Source: Ther Adv Neurol Disord. 2023 Dec 26;16:17562864231213240. doi: 10.1177/17562864231213240 (PMC10752078; doi:10.1177/17562864231213240)
Supplement: sj-docx-1-tan-10.1177_17562864231213240 – Supplemental material for Guideline for the management of myasthenic syndromes [file sj-docx-1-tan-10.1177_17562864231213240.docx]

Tables (1-6) – Supplementary material

Table 1: Overview of scales and scores

| Measure | Domain | Point value | Execution/Interpretation |
| --- | --- | --- | --- |
| MGFA Clinical Classification | Classification to classify clinical features and severity of MG | I-V, higher value corresponds to higher severity  a: Emphasis on extremity musculature  b: Emphasis on oropharyngeal/respiratory musculature | Classification performed by examiner  Initially developed to classify according to historical maximum severity (i.e., a patient with past myasthenic crisis always remains MGFA V) |
| QMG | Quantitative score for objective assessment of disease severity by examination of sentinel muscle groups | 13 items, score 0-3, total score 0-39, higher scores correspond to more severe symptomatology | Survey performed by examiner  Handheld spirometer and vigorimeter required  Change of ≥ 3 points considered clinically significant |
| MG-ADL | Questionnaire to assess the impact of myasthenic syndrome on activities of daily living | 8 items, score 0-3, total score 0-24, higher scores correspond to greater ADL impairment | *Patient-reported outcome*  Change of ≥ 2 points considered clinically significant |
| MG*-*QoLr | Questionnaire to assess the impact of myasthenic syndrome on quality of life (QoL) | 15 items, score 0-4, total score 0-60, higher scores correspond to more severe QoL impairment | *Patient-reported outcome*  No cut-off for clinically significant change defined so far |
| MGC | Composite score developed from key items of QMG, MG-ADL, and MMT (hardly used in original version) | 10 items, different point weighting of the individual items, total score 0-5 | Survey/questioning performed by investigator  Change of ≥ 3 points considered clinically significant |

MG-ADL: Myasthenia gravis Activities of Daily Living Score; MGFA: Myasthenia gravis Foundation of America; MGC: Myasthenia gravis Composite Score; MG-QoLr: revised Myasthenia gravis Quality of Life Score; MMT: Manual Muscle Test; QMG: quantitative Myasthenia gravis Score

Table 2: Cholinesterase inhibitors (AChE-I)

| Substances | Dosage | Side-effects | Contraindications |
| --- | --- | --- | --- |
| **AChE-I** | | | |
| Pyridostigmine bromide (approved) | Unretired:  p. o.: 5-90 mg, onset of action after 45-60 min, maximum effect 3-5 h.  max. 720 mg/d  i. v.: 2-3 mg, 1 mg i. v. corresponds to 30 mg orally.  Onset of action after 5 min, max. 24 mg/d.  i. m.: 2 mg  retarded: 90--180 mg, onset of action after 60 min,  Maximum effect 6-10 h | Stimulation of muscarinic AChR (smooth muscle, glandular secretion):  Abdominal cramps, nausea, vomiting, anorexia, diarrhea, urinary urgency, salivation/tears, sweating, bronchial secretion, accommodation disorders, miosis, bradycardia (rarely AV block), hypotension.  Stimulation of nicotinic AChR (skeletal muscle):  Muscle fasciculations, spasms, muscle weakness (depolarization block).  Differentiation from "cholinergic" crisis (intoxication) | Bronchial asthma, prostatic hypertrophy, decompensated heart failure, recent myocardial infarction, thyrotoxicosis  Stenosis or spasm of the intestinal tract, biliary or urinary tract  Relative contraindications:  Pregnancy, lactation (absolute CI if i. v.). |
| Edrophonium chloride  (Camsilon®; diagnostic purposes only)  alternatively: edrophonium bromide | i. v.: 10 mg, onset of action after 30 sec, maximum effect after 1-2 min | Analogous to pyridostigmine | Analogous to pyridostigmine  Edrophonium test should only be performed in compliance with the contraindications and necessary safety precautions |
| Neostigmine Bromide | p. o. 15 mg (off label)  i. v.: 0.5 mg, onset 5 min  i. m. 1 mg, onset 10-30 min  Maximum effect 2-3 h | Analogous to pyridostigmine | Analogous to pyridostigmine |
| Distigmine Bromide | Maximum daily dose 2 × 5 mg  Maximum effect 12-24 h | Analogous to pyridostigmine | Analogous to pyridostigmine.  Risk of accumulation! Poor controllability |
| Ambenonium chloride (off-label, for bromide allergy) | p. o.: 5-10 mg, onset of action after 60 min, maximum effect after 6-8 h  Maximum 40 mg/d available orally only | Fewer gastrointestinal side effects than pyridostigmine | Analogous to pyridostigmine |

AChE-I: acetylcholinesterase inhibitors; AChR: acetylcholine receptor; AV: atrioventricular; CI: contraindication; i. v.: intravenous; p. o.: oral.

Table 3: Immunotherapies for MG

| Substances | Indication | Efficacy | Dosage | Specific side-effects | Specialties | Checks | Contraindications |
| --- | --- | --- | --- | --- | --- | --- | --- |
| GCS: Prednisone  Prednisolone Methyl-prednisolone | First choice  Initial therapy  Pulse therapy during exacerbation | 2-4 weeks  Effect can be finally assessed after 6 months | 0.5-1.5 mg/kg bw  Prednisone equivalent  Single dose:  10-20 mg/d, increasing by 5-10 mg per week until stable remission is achieved  500-1,000 mg/d for 1-3 days | Weight gain, cushingoid habitus, acne, diabetes, susceptibility to infections, tendency to thrombosis, increase in blood pressure, hypokalemia, edema  Osteoporosis with risk of fractures, aseptic bone necrosis, cataract, glaucoma, mental disorders (euphoria / depressive mood), insomnia, steroid myopathy, promotion of gastric and duodenal ulcers, sexual dysfunction, growth retardation in children. | Initial transient worsening of bulbar symptoms  14 days interval from active vaccination | Blood sugar  Blood pressure  Bone density  Electrolytes, liver values | (relative CI in myasthenic crisis)  Florid bacterial infections, systemic mycoses, manifest gastric and duodenal ulcers, severe osteoporosis, psychiatric diseases, difficult-to-control hypertension, diabetes that has gone off track  In case of osteoporosis: prophylaxis from the very beginning |
| AZA (approved) | First choice Permanent therapy | 6-9 months  Effect can be finally assessed after 18-24 months | 2-3 mg/kg bw  Maintenance dose:  1.5-2 mg/kg bw  Dosage according to laboratory: target value: absolute lymphocyte count 0.6-1.0/nl, without lymphocyte count falling below 0.3/nl | Susceptibility to infections, bone marrow depression (leukopenia thrombopenia, rarely anemia), nausea, vomiting, diarrhea. Fever, hypersensitivity reactions, ideosyncrasia, hepatotoxicity, rarely fever, joint pain, arthralgias, myalgias, alveolitis, pancreatitis, skin exanthema,  rarely lymphoma, melanoma | May be prescribed or continued during pregnancy if indicated.  Breastfeeding: Therapy and full breastfeeding are not mutually exclusive.  In individual cases, a blood count control of the child can be performed if there are corresponding suspicious moments. | Blood count plus differential blood count, liver values (GOT, GPT, GGT, bilirubin, AP), pancreas values (lipase, amylase) and kidney values (creatinine) | Severe infections  Pre-existing liver damage |
| CSA (off-label) | Second choice  Permanent therapy | 1-3 months  Effect can be finally assessed after 6-12 months | 2 (-5) mg/d/kg bw in 2 single doses.  Alternatively, according to valley level: 80-120 µg/l | Hypertension, nephrotoxicity (nephropathy, hyperkalemia), CNS toxicity (tremors, paresthesias, seizures), reversible posterior leukocephalopathy Hepatotoxicity, hirsutism, gingival hyperplasia, secondary neoplasms, infections  Myelosuppression | Note drug interaction | Kidney and liver values Mirror checks | Renal failure  Pregnancy |
| MTX  (off-label) | Second choice  Permanent therapy | 2-6 months  Effect can be finally assessed after 6-12 months | 7.5-15 mg once per week  max. 25 mg once a week as short-term therapy;  in each case in combination with folic acid (5 mg) 24 h after application. | Hepatotoxicity, bone marrow depression, gastrointestinal symptoms, stomatitis, ulcers, exanthem, hair loss, hyperuricemia, renal dysfunction, cystitis, pulmonary fibrosis, cutaneous vasculitis, photosensitivity, psychiatric disorders, osteopathy. | Parenterally preferred due to uncertain oral bioavailability | Liver, kidney and blood count checks  Regular lung function check-up | Pre-existing liver impairment, obesity, alcoholism, bone marrow depression, renal insufficiency, florid gastrointestinal ulcers  Pregnancy |
| MMF  (off-label) | Second choice  Permanent therapy | 2–12 months  Effect can be finally assessed after 12-18 months | 0,5–3 g/d  Usually 2 × 1g/d  Children 600mg/m2 KOF in 2 single doses, max. 2 x 1g  (trough level control) | Gastrointestinal symptoms (nausea, vomiting, diarrhea, ulcers, GI bleeding), leukopenia, anemia, thrombocytopenia, infections (including sepsis and opportunistic infections, candidiasis, herpes simplex, herpes zoster)  Risk of lymphoma under long-term therapy | Reimbursable after failure or intolerance of AZA  Trough level controls | Liver, kidney value and blood count checks | Pregnancy: safe contraception up to 6 months after discontinuation of therapy |
| Tacrolimus  (off-label) | Second choice  Permanent therapy | 1-3 months  Effect can be finally assessed after 6-12 months | 0.1-0.2 mg/kg bw in 2 single doses  According to trough level: 3-4.5 µl/l | Hypertension, nephrotoxicity (nephropathy, hyperkalemia), CNS toxicity (tremor, paresthesias, seizures), encephalopathy (posterior E.), hepatotoxicity, hirsutism, gingival hyperplasia, secondary neoplasms, infections | Induction or blockade of CYP3A4 metabolism to interact with other drugs and/or food (such as grapefruit juice)  Trough level determination | Electrolytes, kidney values | Renal failure  Pregnancy |
| CD20-Ab (Rituximab;  off-label) | Intensified therapy (1st choice) | 6 weeks  Effect can be finally assessed after 6 infusion reactions 12 months | 1,000 mg i.v. on days 1 and 15, then every 6-12 months (individual adjustment of cycle intervals), usually at a reduced dose of e.g. 500 mg or 375 mg/m2 bs i.v. once.  Children: 375 mg/m2 bs | Infusion reactions within 24 hours after application, infections (upper and lower respiratory tract, urinary tract infections), toxic epidermal necrolysis (Lyell syndrome), Stevens-Johnson syndrome,  Hypogammaglobulinemia  Hepatitis B reactivation | Reduced vaccination response likely under therapy, titer control or revaccination if necessary | CD19 B cell status  Hepatitis serology  IgG and IgM level |  |
| Eculizumab | Intensified therapy (1st choice) of generalized refractory AChR-Ab-positive MG | 1-4 weeks  Effect can be finally assessed after 3-6 months | Week 1-4:  900mg weekly i. v.  From week 5:  1,200 mg every 2 weeks i. v. | Infusion reactions, allergic reactions, or  Hypersensitivity reactions  Tendency to infections, especially meningococcal infections | Previous vaccination against meningococci (and pneumococci if necessary) obligatory or chemoprophylaxis | Blood count | Intolerance to eculizumab or other components or in acute meningococcal infection (relative CI for acute bacterial infections) |
| Ravulizumab | Add-on therapy (first-line) of generalized AChR-Ab-positive MG with high disease activity. | 10 weeks  Effect can be finally assessed after 3-6 months | Day 1: Body weight adapted (40-60 kg, 60-100 kg, >100 kg).  Dosage  (2400, 2700 or  3000 mg) i. v.  Day 15 and every 8 weeks thereafter:  Body weight adapted (40-60 kg, 60-100 kg, >100 kg).  Dosage  (3000, 3.300  or 3,600 mg) i. v. | Infusion reactions, allergic reactions, or  hypersensitivity reactions  Headache, nausea, diarrhea, fever, fatigue  Tendency to infections, especially nasopharyngitis, upper respiratory tract infections, urinary tract infections (caution: meningococcal infection)  (caution: meningococcal infection) | Previous vaccination against meningococci (and pneumococci if necessary) obligatory or chemoprophylaxis | Blood count | Intolerance to ravulizumab or other components or in acute meningococcal infection (relative CI for acute bacterial infections) |
| Efgartigimod | Add-on therapy (first-line) of generalized AChR-Ab-positive MG with high disease activity | 4 weeks  Effect can be finally assessed after 2 therapy cycles | Standard use: 10 mg/kg bw i. v. 1 x weekly for 4 weeks, then infusion-free interval of at least 4 weeks and, based on clinical assessment, repeat 4-week cycle  (if necessary, also in flexible intervals) | Headache, nausea, diarrhea  Tendency to infections, especially nasopharyngitis, upper respiratory tract infections, urinary tract infections |  | Blood count IgG-Level | Intolerance to efgartigimod or other components, acute bacterial infections, IgG deficiency |
| Cyclophosphamide (off-label) | Reserve therapy for individual cases | 2–6 months | Pulse therapy:  500-750 mg/m2 bs i. v. every 4-8 weeks.  Empirical cumulative maximum dose with justified indication 50-70 g over the course of several years  Immuno/myeloablative therapy: 50 mg/kg bw on 4 days, followed by administration of G-CSF if necessary | Bone marrow depression, gastrointestinal symptoms, cystitis, hair loss, liver, kidney damage, dermatitis, stomatitis, hyperuricemia  Increased incidence of late tumors | Under urothelial protection with Uromitexan,  sufficient hydration when administering | Blood count  Kidney values | Advanced renal failure  Pregnancy  Acute infections |
| IVIG | Myasthenic crisis/acute exacerbation  Intensified therapy | 1-2 weeks  Effect can be finally assessed after 1-3 months | Short-term treatment: 0.4 g/kg bw for 5 consecutive days alternatively 1 g/kg bw for 2 days.  Initially 5 × 0.4 g/kg bw as pulse, thereafter 1 × 0.4 g/kg bw every 4-8 weeks | Allergic reactions  Hypercoagulability | Applicable in case of exacerbation during pregnancy  Exclude IgA deficiency before starting if possible | IgA level |  |
| PE/IA | Myasthenic crisis/ acute exacerbation  Intensified therapy |  | Short-term treatment: myasthenic crisis  6-8 treatments  Maintenance therapy every 4 weeks | Hypotension  Infections  Secondary Ak deficiency syndrome (IgG < 150 mg/dl) | Substitution with human albumin is necessary after each PE. | Coagulation parameters |  |

AChR: Acetylcholine receptor; AZA: Azathioprine; BS: body surface; BW: body weight; CI: contra indication; CSA: Ciclosporin A; G-CSF: Granulocyte colony-stimulating factors; GCS: Glucocorticosteroids; Ig Immunoglobulin; MMF: Mycophenolate-Mofetil; MTX: Methotrexate; i. v.: intravenous; IA: Immunoadsorption; PE: Plasma exchange

Table 4: Medications that may worsen MG

| Substance group/substance | Commentary |
| --- | --- |
| **Relevant risk, myasthenia-enhancing effect; relatively well documented.** | |
| Antibiotics | SPECIAL CAUTION especially in the treatment of aspiration pneumonia! Cephalosporins are considered safe. |
| (1) Telithromycin (macrolide) | Should not be used in MG. "Red Hand Letter": Myasthenia exacerbation due to telithromycin 2003 |
| (2) other macrolides (Erythromycin, Azithromycin, Clarithromycin among others) | Cautious use if no alternatives applicable |
| (3) Fluoroquinolone (Ciprofloxacin, Levofloxacin, Norfloxacin among others) | Avoid, if possible, since 2011 FDA "black box" warning for fluoroquinolones in MG |
| (4) Aminoglycosides (gentamicin, neomycin, tobramycin) | Cautious use if no alternatives applicable. Potential worsening of MG even with topical use (eye drops). |
| **Blockade of neuromuscular signal transmission** | |
| (1) Botulinum toxin | Avoid |
| (2) Quinine | Quinine also in bitter lemonades, medical history: quinine exposure test in MG diagnostics; an immunological effect was also suspected with chloroquine. |
| (3) Magnesium | potentially dangerous in case of i.v. administration, e.g. in case of eclampsia or hypomagnesemia |
| **Immunological MG induction** | |
| (1) Checkpoint inhibitors (Pembrolizumab, Nivolumab, Atezolizumab, Avelumab, Durvalumab, Ipilimumab among others) | Induction and worsening of MG, fatal courses described; close consultation between oncology and neurology |
| (1) D-Penicillamine | Avoid; strong association with MG |
| **Others** | |
| GCS | Standard therapy of MG, but may lead to initial worsening in the first 2 weeks of treatment ("steroid dip"). Education, monitoring, slow dosing especially in case of bulbar/respiratory symptoms |
| Beta blocker | Older reports after i. v. administration; cautious use |
| Procainamide | Cautious use |
| Statins | Cautious use, when indicated, and in the lowest possible dosage |
| Deferoxamine | Cautious use |
| Contrast media containing iodine | Older preparations, modern contrast media seem to be safe |

**Table 5: Symptoms of MG depending on age of manifestation**

| Age of manifestation | Symptoms | Differential diagnosis |
| --- | --- | --- |
| Neonatal period | - reduced fetal movements - "floppy infant" syndrome - weakness in drinking, high palate - muscular hypotonia - sucking and swallowing disorders - weak crying - apnea - respiratory problems - AMC | - transient form of autoimmune-induced MG. - "Floppy Infant" with other causes such as congenital neuropathies, myopathies and CNS disorders - other causes of congenital contractures - cerebral seizures in the neonatal period - epilepsy syndromes - epilepsy in the context of superordinate diseases |
| After the neonatal period at any age | - ptosis, strabismus, external ophthalmoplegia, high palate - muscle weakness, hypomimia - bulbar symptoms - nasal speech - contractures - stress intolerance - apnea - crises with respiratory distress and ventilatory insufficiency - recurrent apnea and bulbar symptoms at weekly intervals | - autoimmune-mediated MG - other diseases with congenital contractures - other hereditary neuromuscular diseases - Mitochondriopathies - CNS diseases - Epilepsy as a symptom of systemic disease |

AMC: Arthrogryposis multiplex congenita; CNS: central nervous system; MG: Myasthenia gravis

**Table 6: Drug therapy of LEMS**

| Substances | Dosage | Side-effects | Contraindications |
| --- | --- | --- | --- |
| Amifampridine-Phosphate  (3,4-DAP/ Amifampridine) | maximum daily dose 60 mg  (slow dosing!) (according to expert knowledge also usable up to 100 mg/day) | Perioral paresthesias, finger, abdominal pain, diarrhea, nausea, epileptic seizures, cardiac arrhythmias.  Drug interactions with numerous medications (excerpt):  SSRIs, fluoroquinolones, antimalarials, tricyclics, GCS, atypical neuroleptics, opioids, theophylline, sedating antihistamines | known allergy, active epilepsy, poorly controlled bronchial asthma  concomitant therapy with drugs with a very narrow therapeutic window, tendency to prolong the QT time in the ECG, use of sultopride  congenital QT syndromes  cave in patients with renal or hepatic insufficiency |
| Pyridostigmine | see ▶ Tab. 2 |  | |
| Immunotherapy | see ▶ Tab. 3 |  |  |

DAP: diaminopyridine; ECG: electrocardiogram; GCS: glucocorticosteroids; SSRI: selective serotonin reuptake inhibitors
